# Application of 3D point cloud and visual-inertial data fusion in Robot dog autonomous navigation

**DOI:** 10.1371/journal.pone.0317371

**Published:** 2025-02-11

**Authors:** Hongliang Zou, Chen Zhou, Haibo Li, Xueyan Wang, Yinmei Wang

**Affiliations:** State Grid Zhejiang Electric Power Supply Company Taizhou Branch, Taizhou, China; National University of Sciences and Technology NUST, PAKISTAN

## Abstract

The study proposes a multi-sensor localization and real-timeble mapping method based on the fusion of 3D LiDAR point clouds and visual-inertial data, which addresses the issue of decreased localization accuracy and mapping in complex environments that affect the autonomous navigation of robot dogs. Through the experiments conducted, the proposed method improved the overall localization accuracy by 42.85% compared to the tightly coupled LiDAR-inertial odometry method using smoothing and mapping. In addition, the method achieved lower mean absolute trajectory errors and root mean square errors compared to other algorithms evaluated on the urban navigation dataset. The highest root-mean-square error recorded was 2.72m in five sequences from a multi-modal multi-scene ground robot dataset, which was significantly lower than competing approaches. When applied to a real robot dog, the rotational error was reduced to 1.86°, and the localization error in GPS environments was 0.89m. Furthermore, the proposed approach closely followed the theoretical path, with the smallest average error not exceeding 0.12 m. Overall, the proposed technique effectively improves both autonomous navigation and mapping for robot dogs, significantly increasing their stability.

## 1. Introduction

### 1.1. Research background

The fields of automation and intelligence have increasingly focused on mobile robots, particularly robot dog (RD) with high flexibility and maneuverability [1]. RD is capable of performing a variety of tasks in complex and dynamic environments, including search and rescue, patrol monitoring, and household assistance. The successful completion of these tasks relies heavily on the RD’s autonomous navigation abilities. This means that in unfamiliar or rapidly changing environments, the RD must be able to sense its surroundings, determine its own position, plan a path, and avoid obstacles in order to achieve its objectives. 3D point cloud technology is an efficient way to obtain environmental information. It collects distance information from surrounding objects using sensors such as lidar and stereo vision to generate a highly accurate 3D environmental model [2]. The three-dimensional data provides spatial information for the RD, enabling it to perform precise positioning and path planning in three-dimensional space. However, as application scenarios become more complex, a single three-dimensional point cloud may not meet the comprehensive needs of autonomous navigation for RD. The RD use localization, simultaneous localization, and mapping (SLAM) technologies to operate in open and semi-closed environments and meet diverse task demands [3]. One of the key components of RD autonomous navigation (RDAN) is positioning and SLAM technology, which creates a precise real-time map of the surroundings using the installed sensors’ perception of the RD’s working environment and aids in path planning to realize the RDAN in a variety of working scenarios [4]. While the related moving object state estimation methods need to reset different parameters in different scenarios, this limits the algorithm’s ability to adapt in real-world situations. The current research for RDAN in unknown environmental scenarios typically carries multiple sensors, but neglects the multi-level fusion between sensors as well as mutual assistance. Moreover, conventional single sensors or basic algorithms have failed to meet the requirements of RD for accurate positioning and path planning in such situations. Hence, it is of great practical significance and application value to offer an autonomous navigation technology that can ensure high-precision positioning and effective path planning in intricate environments.

### 1.2. Research motivation and purpose

The motivation for this research is to address the challenges of degraded positioning accuracy and realistic reconstruction that RD face when navigating autonomously in complex environments. Since it is difficult for a single sensor data and a simple algorithm to meet the needs of RD for accurate positioning and path planning in a changing environment, the research aims to improve the navigation accuracy and adaptability of RD by developing a multi-sensor positioning and reality reconstruction method based on three-dimensional LiDAR point cloud reality fusion technology. In addition, the research also pursues the real-time and robustness of the algorithm to ensure that the RD can quickly respond to environmental changes and make accurate navigation decisions.

### 1.3. Research innovation

Based on the above, the study recommends a multi-sensor localization and realistic reconstruction (RR) technique that utilizes 3D-LIDAR point cloud reality fusion (3D-LPCRF) to enhance the localization precision of RD in complex environments, therefore improving its capacity to attain autonomous navigation and yield valuable outcomes. The innovation of the research is to integrate multimodal data of LiDAR, camera and inertial measurement unit (IMU) to achieve efficient data fusion and time synchronization, and to optimize the system architecture to improve the real-time and robustness of the algorithm. In addition, a novel multi-stage fusion technique is proposed to optimize the system by factor graph optimization and three-dimensional point cloud information to achieve high-precision positioning and autonomous navigation.

### 1.4. Research content and road map

**Fig 1:**
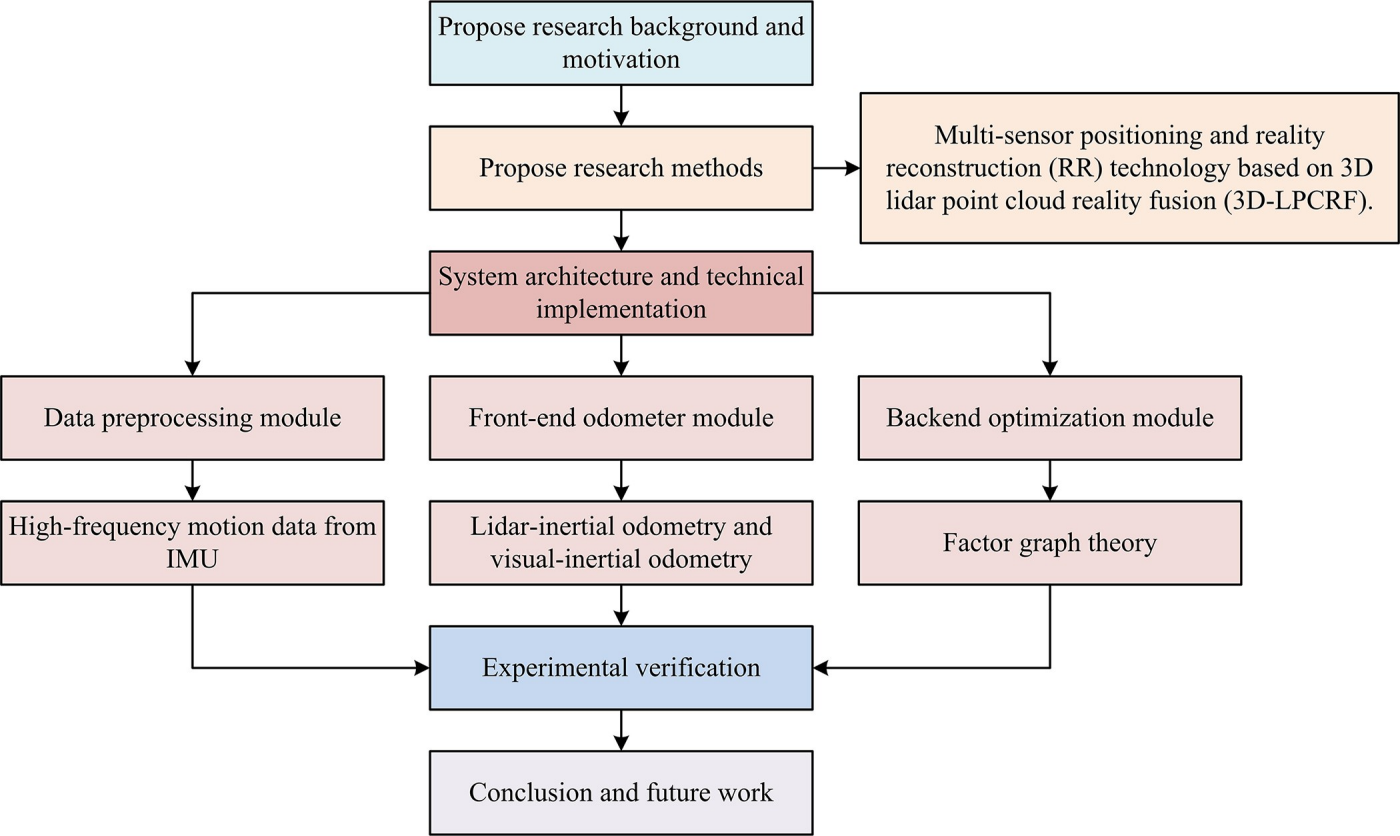
The technical route of the study.

The entire study is broken down into four parts. The first part summarizes and discusses the various multi-sensor fusion localization techniques currently used for robots. The study of 3D-LPCRF technology for RDAN is covered in the second section, along with an analysis of the system architecture and its three internal parts. The verification of the research approach is the third component. A synopsis of the entire essay appears in the fourth section. The technical route of the study is shown in Fig 1.

## 2. Related work

### 2.1. Research status

The development prospect of robot navigation is promising, and it has realized a wide range of applications in many fields. With the continuous advancement of artificial intelligence and machine learning technology, robot navigation systems have become intelligent and autonomous, capable of performing complex tasks independently. J. de Heuvel et al. proposed a spatio-temporal attention pipeline based on two-dimensional lidar sensor readings to enhance the navigation function for predictive navigation of robots in dynamic indoor environments. Through a new LiDAR state representation, the method emphasized the recognition of dynamic obstacles rather than static obstacles. This method also used the attention mechanism to realize the selective scene perception across time and space [5]. M. Shahriari et al. proposed a new navigation method for the safe and efficient navigation of heterogeneous multi-mobile robots. By calculating the collision time and designing a new collision avoidance controller based on it, the robot dynamics (mass and inertia) were integrated into the motion control algorithm to achieve safe navigation [6]. H. Xing et al. proposed a self-localization system based on multi-sensor fusion to address the localization problem of small robots in GPS-denied and structured underwater environments. The system utilized low-cost sensors to achieve high-precision positioning. The extended Kalman filter was utilized to synthesize multi-source information from IMUs, optical flow, pressure sensors, and ArUco markers through multi-sensor information fusion. This enabled the robot to achieve highly accurate positioning [7]. U. Ali et al. proposed a multi-sensor fusion method based on neural networks under predictive coding for the underwater self-localization problems of underwater robots, which effectively optimized the fusion and approximation of noisy sensor information, thus improving the self-localization capability of underwater robots [8]. M. Osman et al. proposed an efficient and versatile multi-sensor fusion method based on mobile horizon estimation for the problems related to the positioning of intelligent vehicles and mobile robots. The study constructed a multi-sensor fusion framework based on it, which effectively improved the accuracy of mobile robot positioning [9]. M. Yang et al. addressed the localization problems of industrial robots in practical operations by fusion of ultrasonic sensors, IMUs, and vision sensors were fused, thus improving robot positioning accuracy while reducing robot positioning drift [10].

In addition, V. B. Hoang et al. sought to address the issue of ensuring the safe and socially acceptable approach of mobile service robots to humans or crowds in a dynamic social environment. To this end, the study proposed a navigation framework for human proximity robots. The potential approach posture was estimated by using a dynamic social region model. In addition, a goal-oriented time elastic band model was used to select the socially optimal approach posture and estimate the socially optimal trajectory of the robot. It improved the safe and effective approach of the robot to individual humans or human groups while ensuring human comfort and socially acceptable behavior [11]. Aiming at the safe and efficient navigation of heterogeneous multi-mobile robots in dynamic environments, M. Shahriari et al. proposed a distributed navigation method that integrates dynamics. By calculating the collision time and designing a new nonlinear controller, the robot optimized the integration of dynamics in motion control algorithms and improved the safety and efficiency of navigation [12]. T. Shan et al. proposed a tightly coupled LiDAR vision-inertial odometry (VIO) framework LVI-SAM for real-time state estimation and map construction with high accuracy and robustness. The framework improved the accuracy and robustness of vision-inertial systems (VIS) and LiDAR inertial systems (LIS) through smoothing and mapping [13]. To improve the positioning accuracy and system robustness of robots and autonomous vehicles in complex environments, J. Lin et al. proposed a novel LiDAR-inertial-vision sensor fusion framework R3 LIVE. By integrating data from LiDAR, IMUs, and vision sensors, the framework enabled accurate, dense, 3D, RGB color maps of the environment to be reconstructed in real time. The R 3 LIVE consisted of two subsystems: LiDAR-inertial odometry (LIO) and VIO. The LIO subsystem (FAST-LIO) built the geometry of the map using LiDAR and IMU measurements, while the VIO subsystem fused visual data directly and efficiently by minimizing frame-to-map photometric errors to add texture to the map [14].

### 2.2. Problems and limitations

While some progress has been made in the fields of autonomous navigation and multi-sensor fusion, there are still significant limitations. One major issue is the lack of adaptability in dynamic and unpredictable environments, which makes it difficult to achieve effective dynamic scene mapping and obstacle avoidance. Secondly, sensor fusion technology that relies on complex data association and optimization increases the computational burden and affects the real-time performance of the system. Additionally, the method for estimating the state of moving objects requires parameter adjustment for different scenarios, which limits the algorithm’s generalizability and practical application. Current research often overlooks multi-level fusion and mutual support between sensors, which can negatively impact the overall performance and robustness of the system. In addition, many methods have only been tested in predefined scenarios, and their effectiveness in unknown environments and real-time changing conditions has not been fully verified. Finally, the noise and incompleteness of a single 3D point cloud also limits the accuracy of environmental modeling and positioning.

### 2.3. Innovation and significance

Therefore, the proposed multi-sensor multilevel fusion RD localization and real-scene fusion technique is innovative, and its internal optimization by factor graph optimization and the use of 3D LIDAR point cloud information optimizes the system to achieve high-precision localization and autonomous navigation. At the same time, the research content is to construct a pollution-free large-scale static 3D scene map for unmanned mobile RD working in complex dynamic scenes, and further construct a semantic map of surrounding scenes based on semantic information, effectively improving the autonomous navigation ability of RD, creating huge economic benefits for society, and also providing assistance in liberating people from labor-intensive work and work with safety hazards.

At the same time, the practical results of the research can be used to construct a pollution-free large-scale static 3D scene map for unmanned RD working in complex dynamic scenes, and further construct a semantic map of surrounding scenes based on semantic information, thereby improving the autonomous navigation ability of RD and performing precise unmanned operations in complex work scenes, thereby reducing labor costs and improving production and living efficiency, while creating enormous economic benefits for the whole society and freeing humanity from labor-intensive and safety hazards. Current research on multi-sensor positioning and scene reconstruction aims to integrate multi-modal data in observation models to improve positioning accuracy. However, it often neglects multi-level fusion between sensors and mutual assistance among them. The proposed methods are innovative in both theory and practice. Meanwhile, the research method used in the study on the quadruped RD can also be applied to wheeled robots due to their high generalization ability.

## 3. 3D-Lpcrf technology for RDAN

### 3.1. Analysis of positioning and RR system architecture in RDAN

Quadruped RD is widely used in practical industrial scenarios or complex environments due to their strong terrain adaptability, high degrees of freedom, flexible motion, and ability to climb stairs. However, their multi-sensor approach to localization and navigation has perceptual shortcomings. This study proposes a system for automatic navigation of an RD using a multi-sensor localization and RR method based on 3D-LPCRF. The system is constructed using multi-sensor RD positioning and scene reconstruction methods and consists of three core modules: a data pre-processing module, a front-end mileage measurement module, and a back-end optimization module. The data pre-processing module utilizes high-frequency motion data from the IMU to correct RD motion distortion under 3D-LPCRF technology and achieve time synchronization between the camera and lidar sensors. Additionally, the lidar and camera are calibrated to achieve accurate fusion of LiDAR point cloud data and image data, which complement each other. The multi-sensor lower RD positioning and SLAM system in this study includes lidar, monocular camera, and IMU sensors. The system utilizes the multi-modal data collected by these sensors as input. The study employs IMU pre-integration technology and data pre-processing to rectify lidar point clouds, ensuring synchronization and accuracy of IMU and 3D point cloud data. Subsequently, feature extraction and factor graph optimization are combined to achieve high-precision data fusion and enhance the RD’s autonomous navigation capability in complex environments.

In unknown environments, the autonomous navigation of RD mainly relies on localization and real-scene reconstruction, which aims to perceive and reconstruct the surrounding real-scene in real time, so as to obtain the position of RD in the real-scene, and thus optimize the path to achieve navigation [15,16]. It is considered that the RD’s ontological 3D coordinate system in the real scene coincides with the IMU coordinate system, so its localization is equivalent to estimating in real time the relative position and attitude relationship between the IMU coordinate system and the fixed world coordinate system. The three types of sensors utilized in multi-sensor fusion systems exhibit disparate acquisition frequencies. In this case, the data acquisition frequency of the IMU is significantly higher than that of the monocular camera and the LIDAR. Therefore, it is imperative to determine the optimal state of the system to ensure that the output frequency remains synchronous with that of the LIDAR. When LIDAR acquires a 3D LIDAR point cloud for a particular frame, the system state of the RD for that frame is set with the correlation transformation matrix expression of the IMU for that frame with respect to the world coordinates as shown in Eq (1).


$$\{\begin{array}{c} p_{j}=[x_{A_{j}}^{M},\gamma_{A_{j}}^{M},y_{A_{j}}^{M},b_{a_{j}},b_{\varpi j}]\\ \psi_{A_{j}}^{M}=[\begin{array}{cc} T_{A_{j}}^{M} & x_{A_{j}}^{M}\\ 0 & 1 \end{array}] \end{array}$$ (1)

**Fig 2:**
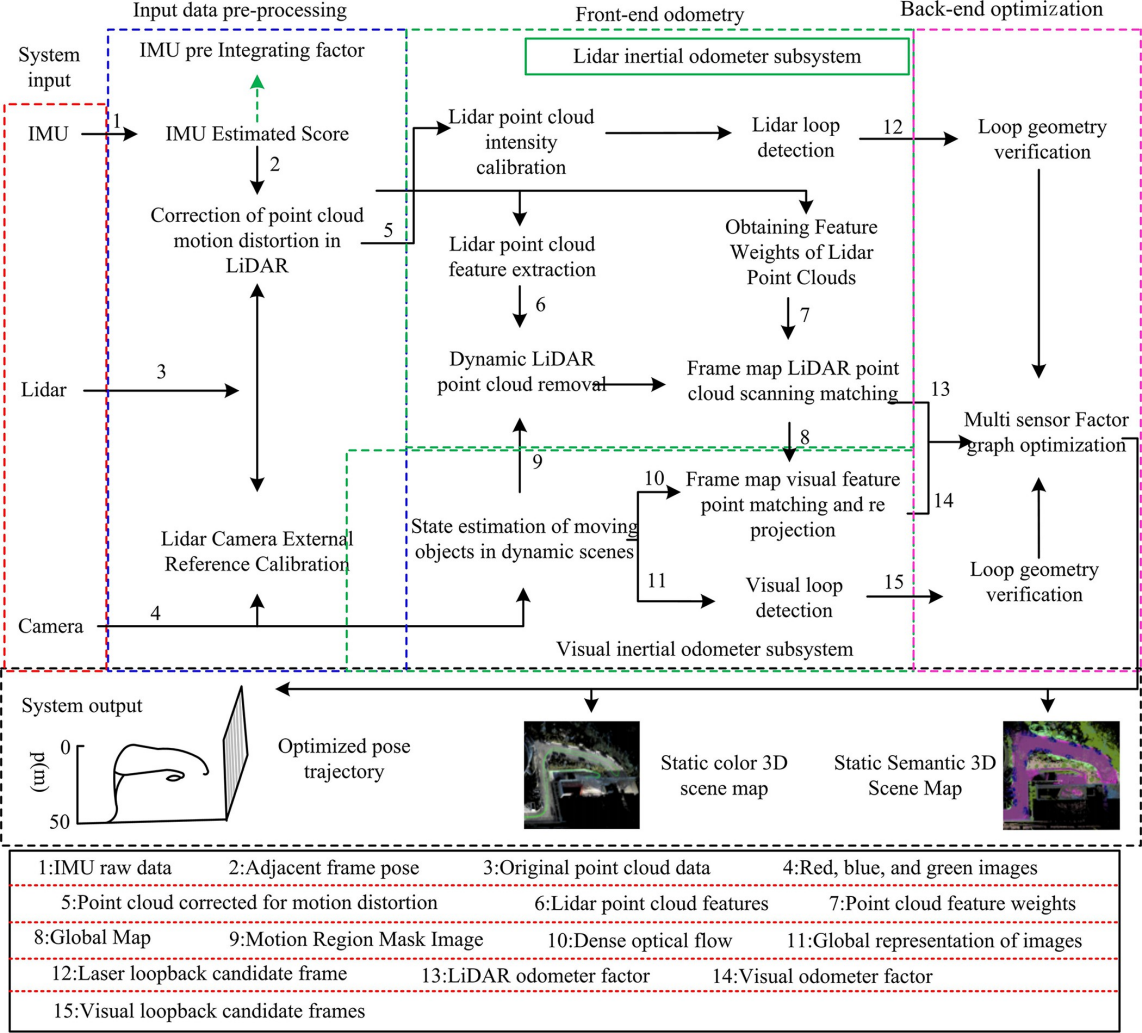
System architecture for localization and scene reconstruction methods in autonomous navigation of RD based on multi-sensor multi-level fusion.

In Eq (1), *p*_*j*_ denotes the collection state of the system at frame *j*. $x_{A_{j}}^{M}$ denotes the position. $\gamma_{A_{j}}^{M}$ denotes the velocity. $y_{A_{j}}^{M}$ denotes the direction. $b_{a_{j}}$ denotes the measurement bias of the accelero-meters in the IMU. $b_{\varpi j}$ denotes the measurement bias of the gyroscopes in the IMU. $\psi_{A_{j}}^{M}$ denotes the correlation transformation matrix of the IMU in frame *j* with respect to the world coordinates. $T_{A_{j}}^{M}$ denotes the correlation rotation matrix corresponding to the $x_{A_{j}}^{M}$. *A* denotes the matrix in the IMU coordinate system. *M* denotes the matrix in the world coordinate system. Eq (1) can provide a basis for the ensuing autonomous navigation system design concerning RD. Accordingly, Fig 2 represents the system design of the suggested multilevel fusion of multiple sensors for localization and SLAM techniques in RDAN [17].

In Fig 2, the system is divided into three main modules, which are input data pre-processing, front-end odometry, and back-end optimization. The initial data pre-processing involves integrating IMU, correcting motion distortion in LiDAR point clouds, and adjusting the external parameters of the LiDAR camera. In the initial setup stage of the system, the LIDAR-camera external parameter online correction method is employed to rectify the external parameters between the two types of sensors in real time. This is done to guarantee the precise integration of the LiDAR point cloud data and the camera image data, and to incorporate additional information such as colors, textures, semantics, and motion states into the point cloud. The front-end of the system consists of a LIO and a VIO The former extracts three types of feature points, namely, points, lines and surfaces, from the corrected point cloud, and constructs the frame-local map scanning matching error function and distance factor based on each feature point. The latter achieves the recognition of LiDAR point clouds in the field of view through the fusion of LIDAR and image data. The method utilizes the LiDAR point cloud’s projection points on to the image as the image’s feature points. It traces the feature points between the images via the dense optical flow and formulates the reprojection error function from the matched image feature points. On this basis, it is proposed to fuse visual loop closure and laser loop closure as a decision layer to improve the detection accuracy of loop closure coefficients in complex dynamic real-world scenes. From a physical perspective, the positioning and scene reconstruction system using multi-sensor fusion inputs point cloud data collected by LIDAR, image data collected by camera memorizes acceleration and angular velocity data collected by IMU. Moreover, the system outputs pose trajectories of RD and static 3D scene maps with color and semantic information. The multi-modal data collected by three sensors in the system is fused at multiple levels and sensors in each module of the system.

The back-end of the system can be outlined as maximum-a-posteriori estimation (MAP) using factor graphs. Factor graph theory is a better inference method than Bayesian networks, which is an undirected graph and consists of variable nodes representing the optimized variables and factor nodes representing the factors [18,19]. Optimizing a factor graph is essentially adjusting the values of each variable to maximize the product of its factors. The MAP inference problem in factor graphs is equated to a class of nonlinear least squares problems under the assumption of a Gaussian noise model. Among them, the variable nodes are the system state variables, and the factors connecting the variable nodes include the IMU pre-integration factor, the LIDAR odometry factor, the visual odometry factor, and the loop closure factor. On this basis, the factor graph is improved by introducing new state variables, making them satisfy certain constraints and adding them to the factor graph as new variable nodes, and when a new node is added, the factor graph is improved by using a Bayesian tree. Among them, the more important of the input data pre-processing module is the IMU pre-integration analysis and correction of the actual motion aberrations of the LIDAR point cloud with the sensor synchronization. Modifications are made to some symbols in the formula based on reference to previous literature due to the consideration of studying practical scenarios and needs. The IMU measurement model is expressed as shown in Eq (2) [20].


$$\{\begin{array}{c} {\tilde{a}}^{A}=\psi_{W}^{A}(a^{W}-h^{W})+b_{a}+n_{b}\\ {\tilde{\upsilon }}^{A}=\upsilon^{A}+b_{\varpi }+n_{\varpi } \end{array}$$ (2)

In Eq (2), the IMU model is reliable. ${\tilde{\alpha }}^{A}$ represents the true value of linear acceleration in a body coordinate system {*A*}. $\psi_{W}^{A}$ denotes the associated rotation matrix from the actual world coordinate system to the IMU coordinate system. *α*^*W*^ represents the actual angular velocity true value of IMU at world coordinate {*W*}. *h*^*W*^ denotes the associated gravitational acceleration in world coordinates. *b*_*a*_ denotes the zero bias of the accelerometer. *n*_*b*_ denotes the associated measurement noise of the accelerometer. ${\tilde{\upsilon }}^{A}$ denotes the angular velocity of the body. *υ*^*A*^ denotes the true value of the actual linear acceleration of the IMU in the body coordinate system. $b_{\varpi }$ denotes the zero bias of the gyroscope. $n_{\varpi }$ denotes the associated measurement noise of the gyroscope. Eq (2) can provide ideas for subsequent IMU pre-integration calculations. When the IMU is fused with other sensors, integration of the kinematic model of the IMU can yield relative motion estimates between two adjacent frames of the LIDAR point cloud. However, the method is contingent upon the system state. Consequently, when the state parameters obtained by optimization are changed, it is necessary to reintegrate all IMU observations within each frame to obtain a revised inter-frame relative motion estimate. For repeated IMU integration, it will increase the computational complexity, so pre-integration is proposed to solve the problem effectively. The main idea of pre-integration is to program the integration terms in the original integration formula from the attitude relative to the world coordinate system to the body coordinate system. The expression of the linear acceleration and angular velocity between the two neighboring moment matrices in the IMU coordinates, with time intervals after applying the median method, is demonstrated in Eq (3). This is provided with the IMU measurements in the coordinates of any two neighboring moments between two adjacent frames [21].


$$\{\begin{array}{c} a=\frac{1}{2}(\left(y_{A_{t}}^{A_{j}}-b_{a_{j}})+y_{A_{t+1}}^{A_{j}}({\tilde{a}}^{A+1}-b_{a_{j}}\right))\\ \psi =\frac{1}{2}(\left({\tilde{\psi }}^{A_{t}}-b_{\varpi_{j}})+({\tilde{\psi }}^{A_{t+1}}-b_{\varpi_{j}}\right)) \end{array}$$ (3)

In Eq (3), *a* denotes the linear acceleration. *Ψ* the angular velocity. *t* the time point. *A*_*t*_ and *A*_*t*+1_ the matrix values of two adjacent moments. To calculate the IMU pre-integration value at time *A*_*t*+1_, it is first necessary to calculate the IMU pre-integration value at time *A*_*t*_, as indicated by Eq (3). The corresponding expression is shown in Eq (4).


$$\{\begin{array}{c} \eta_{A_{t+1}}^{A_{j}}=\eta_{A_{t}}^{A_{j}}+\mu_{A_{t}}^{A_{j}}+\rho t+\frac{1}{2}a\rho t^{2}\\ \mu_{A_{t+1}}^{A_{j}}=\mu_{A_{t}}^{A_{j}}+a\rho t^{2}\\ y_{A_{t+1}}^{A_{j}}=y_{A_{t}}^{A_{j}}⊗[\begin{array}{c} 0\\ \psi \rho t/2 \end{array}] \end{array}$$ (4)

In Eq (4), *η* and *μ* denote the pre-integration values of IMU. *ρt* denotes the time interval between *A*_*t*_ and *A*_*t*+1_. ⊗ denotes quaternion multiplication. Therefore based on Eq (4) the pre-integration value between *A*_*t*_ and *A*_*t*+1_C can be obtained and its expression is shown in Eq (5) [22].


$$\zeta_{A_{j+1}}^{A_{j}}=[\eta_{A_{j+1}}^{A_{j}},\mu_{A_{j+1}}^{A_{j}},y_{A_{j+1}}^{A_{j}},b_{a_{j+1}},b_{\varpi_{j+1}}]$$ (5)

In Eq (5), $\zeta_{A_{j+1}}^{A_{j}}$ denotes the pre-integrated value between *A*_*t*_ and *A*_*t*+1_. The introduction of IMU pre-integration can not only effectively improve the operational efficiency of the actual calculation, but also provide a constraint for the factor graph, i.e., the IMU pre-integration factor, whose expression is shown in Eq (6).


$$d_{A}(\zeta_{A_{j+1}}^{A_{j}},x_{j},x_{j+1})=[\begin{array}{c} d_{p}\\ d_{\gamma }\\ d_{q}\\ d_{b_{a}}\\ d_{b_{\varpi }} \end{array}]=[\begin{array}{c} q_{A_{j}}^{W-1}(p_{A_{j+1}}^{W}-p_{A_{j}}^{W}-\gamma_{A_{j}}^{W}\Delta t-\left(h^{W}\Delta t^{2}\right)/2)-\eta_{A_{j+1}}^{A_{j}}\\ y_{A_{j}}^{W-1}(\gamma_{A_{j+1}}^{W}-\gamma_{A_{j}}^{W}-h^{W}\Delta t^{2})-\mu_{A_{j+1}}^{A_{j}}\\ 2{\left[x_{A_{j+1}}^{A_{j-1}}⊗(p_{A_{j}}^{M-1}⊗p_{A_{j+1}}^{W})\right)}_{xyz}\\ b_{a_{j+1}}-b_{a_{j}}\\ b_{\varpi_{j+1}}-b_{\varpi_{j}} \end{array}]$$ (6)

**Fig 3:**
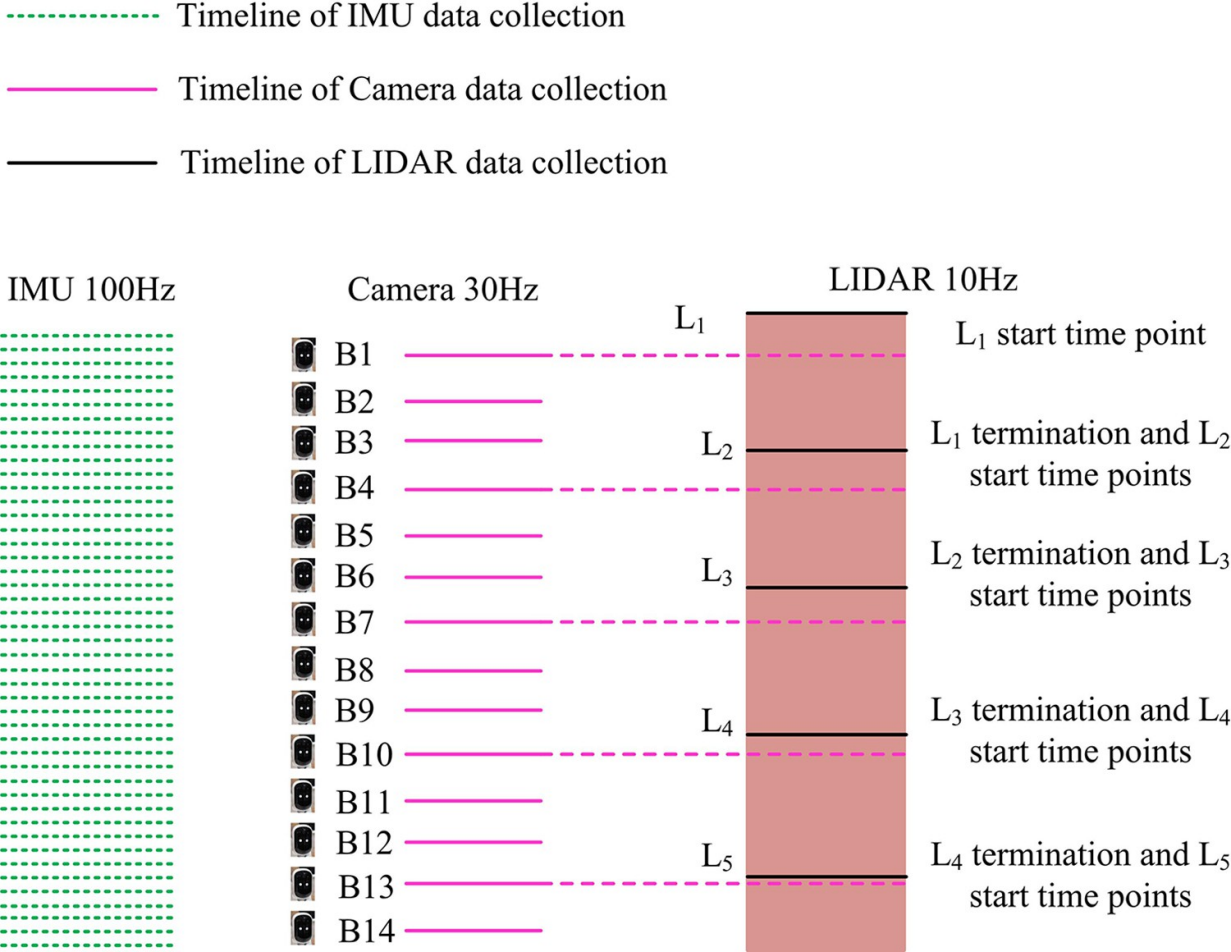
Schematic diagram of time stamp synchronization between LIDAR and camera in a multi-sensor system.

In Eq (6), [∙]_*xyz*_ denotes the imaginary part of the quaternion. By synthesizing Eqs (3) to (6), the IMU pre-integration can be calculated. Compared to the real-time acquisition of devices such as IMUs and cameras, the mechanical rotating LIDAR uses the built-in motor rotation to scan into 360 degrees, and after completing one scan, the collected data is accumulated into a point cloud image before the next scan, which leads to its observation is not carried out at the same time. At high velocities, LiDAR point clouds have the potential to cause considerable distortion in motion. Since LIDAR can provide the timestamp data for each point, the position data provided by the IMU is used to obtain the position of each point cloud through linear interpolation. Then, all laser points in a full frame of the original laser point cloud are translated to the scan start or end time of the current frame, allowing the motion distortion in the current frame’s point cloud to be corrected. Among them, the schematic diagram of LIDAR and camera timestamp synchronization in the multi-sensor system is shown in Fig 3.

In Fig 3, the fusion of point clouds for localization and real-world reconstruction is proposed to be achieved through the use of three sensors: an IMU, a camera, and a LIDAR. The asynchronous processing of the observation data from the LIDAR and camera is then employed to achieve the joint correction of the observation data from the LIDAR and camera. In addition, unifying multi-modal data into the same state node when performing data fusion is made difficult by the inconsistent acquisition frequency of the camera and LIDAR. Therefore to address this problem after the scan starts, the full LiDAR point cloud is transformed to the camera coordinates closest to the current image. When the full point cloud transformation in the LIDAR scan K2 is applied to the coordinate system closest to the coordinate system where the camera image frame B4 labeled at the start moment is located, the LIDAR and the camera are synchronized in time by correcting for the LIDAR point cloud motion distortion. Moreover, the two sensors are aligned in terms of data acquisition frequency. At the same time, the joint consistency of the three input data from IMU, camera and LIDAR is ensured to be optimal by adding the same number of new nodes and coefficients to the coefficient graph.

### 3.2. Front-end design and back-end optimisation of multi-sensor fusion systems in RD navigation

Based on Fig 2, it is known that the system front-end design starts after the input of the data pre-processing module. In the LIO subsystem of the system front-end, when the system receives a frame of LIDAR point cloud, the original point cloud is corrected by data pre-processing to correct the running distortion. Then the obtained point cloud will be fed into the feature extraction module to extract LIDAR feature points. For any point in the LIDAR point cloud of the frame, the K-dimensional tree is used to find the nearest K points in the sphere with radius D. At the same time, the local linearity of the geometric features in the LIDAR point cloud is analyzed using principal component analysis. The neighborhood and local linearity are expressed as shown in Eq (7) [23].


$$\{\begin{array}{c} \sum ƛ=\frac{1}{ƛ}\sum_{{\tilde{\varsigma }}_{i}\in ƛ}({\tilde{\varsigma }}_{i}-\bar{\varsigma }){\left({\hat{\varsigma }}_{i}-\bar{\varsigma }\right)}^{T}\\ \Omega_{l}=\frac{\kappa_{1}-\kappa_{2}}{\kappa_{1}},\Omega_{Ρ}=\frac{\kappa_{2}-\kappa_{3}}{\kappa_{1}},\Omega_{e}=\frac{\kappa_{3}}{\kappa_{1}+\kappa_{2}+\kappa_{3}} \end{array}$$ (7)

In Eq (7), ƛ denotes the neighborhood, ∑ƛ is the covariance matrix. *ς*_*i*_ denotes any point in the LIDAR point cloud. $\bar{\varsigma }$ denotes. Ω_*l*_, Ω_P_, and Ω_*e*_ denote the local linearity, planarity, and curvature. *κ* denotes the feature values. In total, the study extracted three types of LIDAR point cloud features, thus generating three sub-maps of the local map, i.e., point features, line features, and planar features. In the scanning matching process, various functions have been designed based on relevant feature points. Eq (8) shows the expressions for the three scanning matching functions used in this study, as well as the optimal position estimation expression for the current frame, which is based on the function and the least squares estimation method.


$$\{\begin{array}{c} f_{i}^{\varsigma o\to \varsigma o}={\left\|{\varsigma^{\prime }}_{i}-(\psi_{A_{j+1}}^{W}\varsigma_{i}+t_{A_{j+1}}^{W})\right\|}_{2}\\ f_{i}^{\varsigma o\to li}={\left\|{\hat{\varepsilon }}_{i}\times ({\varsigma^{\prime }}_{i}-(\psi_{A_{j+1}}^{W}\varsigma_{i}+t_{A_{j+1}}^{W}))\right\|}_{2}\\ f_{i}^{\varsigma o\to \varsigma l}={\hat{\phi }}_{i}^{T}\cdot ({\varsigma^{\prime }}_{i}-(\psi_{A_{j+1}}^{W}\varsigma_{i}+t_{A_{j+1}}^{W}))\\ {\mathbb{N}}_{A_{j+1}}^{W*}=\underset{{\mathbb{N}}_{A_{j+1}}^{M}}{\arg\min}\{\sum_{\varsigma_{i}\in E_{\varsigma o}^{A_{j+1}}}g_{i}^{\varsigma o}{\left(f_{i}^{\varsigma o\to \varsigma o}\right)}^{2}+\sum_{\varsigma_{i}\in E_{li}^{A_{j+1}}}g_{i}^{li}{\left(f_{i}^{\varsigma o\to li}\right)}^{2}+\sum_{\varsigma_{i}\in E_{\varsigma l}^{A_{j+1}}}g_{i}^{\varsigma l}{\left(f_{i}^{\varsigma o\to \varsigma l}\right)}^{2}\} \end{array}$$ (8)

In Eq (8), $f_{i}^{\varsigma o\to \varsigma o}$ denotes the point-point distance error. $\psi_{A_{j+1}}^{W}$ and $t_{A_{j+1}}^{W}$ denote the elements in the change of the current frame pose. $f_{i}^{\varsigma o\to li}$ denotes the point-line i.e. error. ${\hat{\varepsilon }}_{i}$ denotes the actual direction vector corresponding to the map point feature. $f_{i}^{\varsigma o\to \varsigma l}$ denotes the point-plane distance error. ${\hat{\phi }}_{i}$ denotes the plane normal vector corresponding to the map point feature. ${\mathbb{N}}_{A_{j+1}}^{W*}$ denotes the optimal pose of the current frame. $g_{i}^{\varsigma o}$ denotes the weight of the point-point distance error. $g_{i}^{li}$ denotes the weight of the point-plane distance error. $g_{i}^{\varsigma l}$ denotes the weight of the point-surface distance error. Therefore, the equation expression of the three weights is shown in Eq (9).


$$\{\begin{array}{c} g_{i}^{\varsigma o}=\frac{\Omega_{e}}{\Omega_{e\max}}\\ g_{i}^{li}=1-\frac{\sum_{{\hat{\varsigma }}_{i}\in ƛ}{\left({\hat{\varsigma }}_{i}-\bar{\varsigma }\right)}^{T}(I-{\hat{\phi }}_{i}^{T}{\hat{\phi }}_{i})({\hat{\varsigma }}_{i}-\bar{\varsigma })}{Kf_{\max}}\\ g_{i}^{\varsigma l}=1-\frac{\sum_{{\hat{\varsigma }}_{i}\in ƛ}{\left[{\hat{\phi }}_{i}({\hat{\varsigma }}_{i}-\bar{\varsigma })\right]}^{T}[{\hat{\phi }}_{i}({\hat{\varsigma }}_{i}-\bar{\varsigma })]}{Kf_{\max}} \end{array}$$ (9)

In Eq (9), Ω_*e* max_ denotes the threshold of maximum curvature, here taken as 0.8. *I* denotes the red, green, and blue (RGB) image. *K* denotes the actual number of points in the neighborhood. *f*_max_ represents the actual maximum threshold for point line distance and point surface distance, here taken as 0.2 m. Currently, the actual optimal bit position value of the LIDAR frames can be taken as the absolute limit of the correlation factor graphs of the absolute limit of the corresponding state node, so there is an associated LIDAR odometry factor expression constructed using LIDAR point cloud matching as shown in Eq (10).


$$d_{A}({\mathbb{N}}_{L_{j+1}}^{W}x_{j+1})=[{\begin{array}{c} p_{A_{k+1}}^{W}-{\tilde{p}}_{A_{k+1}}^{W}\\ 2[{\tilde{q}}_{A_{k+1}}^{W-1}⊗q_{A_{k+1}}^{W}] \end{array}}_{xyz}]$$ (10)

In Eq (10), ${\tilde{x}}_{A_{k+1}}^{W}$ denotes the relevant translational part of the output best-position value. ${\tilde{q}}_{A_{k+1}}^{W}$ denotes the relevant rotational part of the output best-position value. Eqs (7) to (10) analyze the LIO subsystem comprehensively through the formula. VIO is implemented to estimate the state of the system by exploiting the reprojection error between the local map points and the corresponding image points in the camera’s field of view. Distinguishing from the conventional feature-point-based visual odometry method, this method uses the projected points of the LiDAR point cloud on the corresponding image as feature points. In the process of real-world reconstruction, the LiDAR point cloud is transformed to world coordinates according to the attitude parameters of each frame. Then the point cloud is accumulated, so as to construct a global map. In the system data pre-processing module, the accurate fusion of the LIDAR point cloud with the image is utilized so that any feature point in the image can be searched for the corresponding global point in the image.

The proposed "VIO" subsystem and the "LIO" subsystem are two independent subsystems. Furthermore, with the help of the factor graph, the overall system can maintain smooth operation even if the former fails to work properly. With the help of the factor diagram, even if the former malfunctions and does not work properly, the overall system can still maintain smooth operation. In low-light environments, if an abrupt light source triggers a significant change in illumination, resulting in the failure to match the visual odometry or a substantial re-projection error, it may be classified as a visual sensor failure. In such cases, it is recommended to remove the visual sensor from the factor graph during construction. Due to the high complexity of the environment faced by the RD in the actual autonomous navigation, the relevant sensors will be affected by the measurement noise. Therefore, it is inevitable that cumulative errors will occur in the process of real-world localization and reconstruction during autonomous navigation. It will lead to the drift of its positional trajectory, and then make the map constructed by it inconsistent with the true real-world view. Loop closure detection algorithm is introduced to eliminate the cumulative offset deviation. It consists of LIDAR odometry and mapping scan contex (LOAM-SC) using intensity scanning context and visual loop closure detection using 3D point cloud fusion technique, only the latter is described due to space limitation.

**Fig 4:**
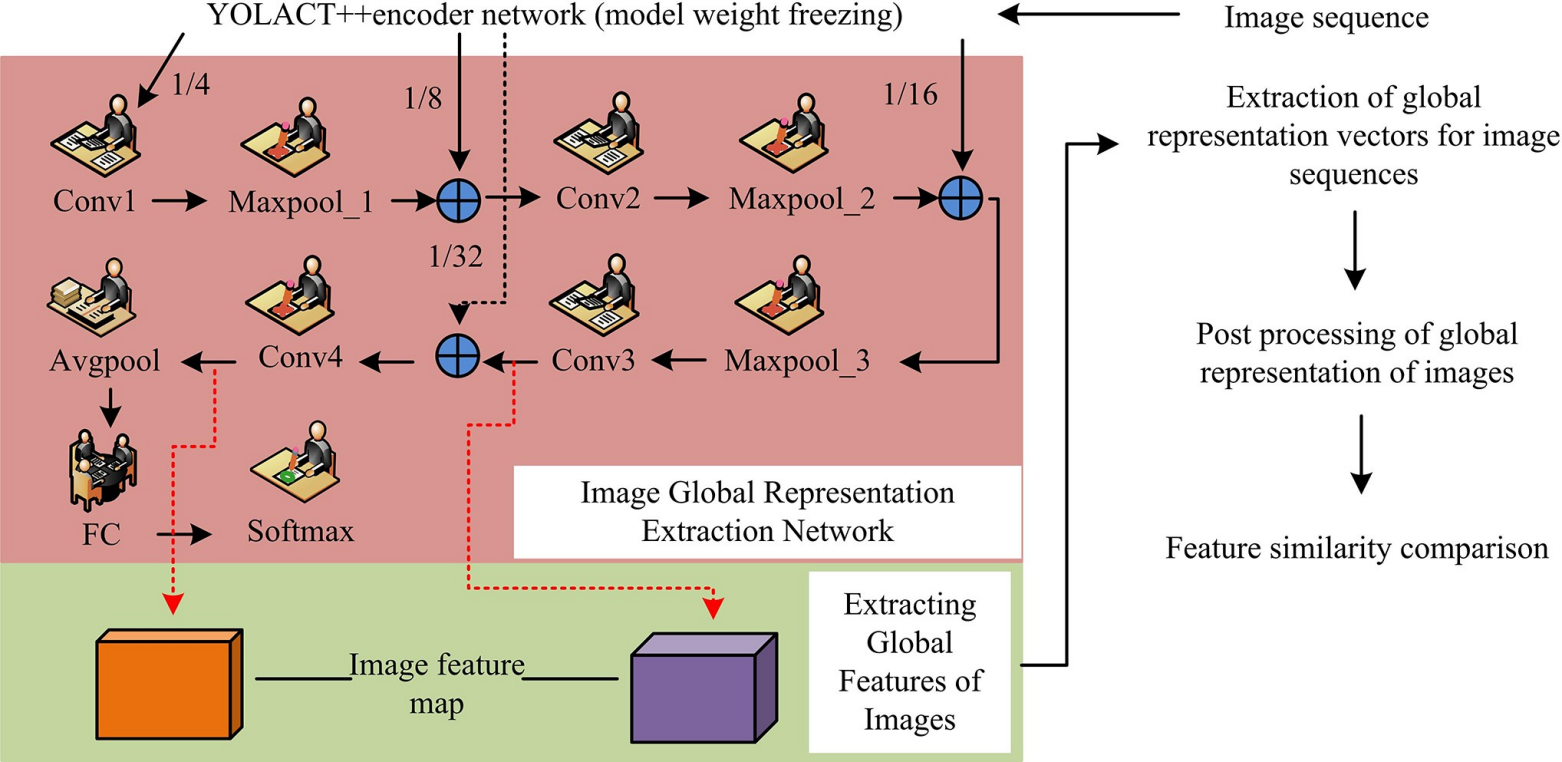
Schematic diagram of loop detection algorithm using image global pointer.

In the proposed research system’s front-end VIO component, the traditional bag-of-words model is not applicable due to the inability to extract feature points from the image. The only viable option is to utilize pixel points provided by the LiDAR point cloud projected onto the image coordinate system as feature points. The study introduces a real scene classification network as an extractor of relevant features of the actual image, which uses global features to characterize the current real scene. At the same time, the candidate frames for loop closure detection are obtained by comparing the feature similarity of the current frame with the feature similarity of the historical frame. Thus, the schematic diagram of loop closure detection algorithm using global representation of image is shown in Fig 4.

Fig 4 shows that the traditional universal network layer has low scene performance when representing special outdoor scenes. To address this issue, the algorithm does not use a universal network layer, and instead focuses on improving the scene representation ability of the network. In Fig 4, the algorithm consists of three parts in total, i.e., extraction of the actual representation vectors of the image, post-processing of the correlation of the global representation vectors, and comparison of the global feature similarity. In the part of extracting the actual representation vectors of the image, the study utilizes the you only look at coefficients ++(Yolact++) in the instance segmentation network for the construction of the correlation feature extractor of the image, and in order to produce the overall features of the image. Moreover, a parallel sub-network is added to the original network structure. The internal structure of this parallel sub-network is shown in Fig 4, containing four convolutional layers, three maximum pooling layers, one average pooling layer, one fully connected layer, and one S-function layer, which is essentially a miniaturized real-world classification network. On this basis, the data set (Places-365) directed towards indoor and outdoor real-view classification is screened for the problem of improving the real-view representation ability of the parallel sub-network and trained accordingly. Fixed weights are used in the learning phase of the Yolact++ encoder. After the network has been trained, a feature vector for an image is constructed using the feature map output from one of the layers of this network.

To construct a global representation vector of an image, it is necessary to flatten a feature map into a feature vector and then concatenate these flattened feature vectors. This transforms the feature tensor output from the network into a one-dimensional image representation vector. The process of loop closure detection involves matching the relevant representation vectors of the current frame with a growing set of historical frame representation vectors. This inevitably increases the computational cost of matching. However, it is necessary for accurate detection. To effectively reduce the cost and improve the actual matching efficiency, a two-stage matching scheme from coarse to fine is proposed. In the coarse matching phase, the actual features of the network are maximally pooled, i.e., each feature map is replaced by its maximum value. Moreover, the original 2048 feature maps are converted into feature vectors of length 2048, which are binarized according to the size of the values, as shown in Eq (11).


$$\lambda_{a}(i)=\{\begin{array}{c} false,\lambda_{v}(i)<0.5\\ true,\lambda_{v}(i)\geq 0.5 \end{array}$$ (11)

In Eq (11), *λ*_*a*_(*i*) denotes the binarized feature vector. *λ*_*v*_ denotes the feature vector of length 2018. In performing the corresponding loop closure detection, the coarse matching image is first used to characterize the m-1 history frames with maximum similarity in the vector query. The feature similarity expression for the coarse matching vector of the query frame and the coarse matching vector of the candidate frame is shown in Eq (12).


$$Z(\lambda_{b}^{q},\lambda_{b}^{e})=\frac{XOR(\lambda_{b}^{q},\lambda_{b}^{e})}{2048}$$ (12)

In Eq (12), *Z* denotes the feature similarity. $\lambda_{b}^{q}$ denotes the coarse matching vector of the query frame. $\lambda_{b}^{e}$ denotes the coarse matching vector of the candidate frame. $XOR(\lambda_{b}^{q},\lambda_{b}^{e})$ denotes the dissimilarity operation of the indirect elements of the two vectors. Secondly, the corresponding fine matching feature vector of the query is used for secondary matching. To prevent any confusion and aid future descriptions, the current frame’s symbol is altered to match the symbol of a historical frame through "fine matching." To reduce the size of the initial image features within the global representation vector, the global feature vectors are converted to the principal component analysis subspace via principal component analysis. The image similarity is then evaluated on this low-dimensional subspace. The practical purpose of loop closure detection is to resolve the cumulative errors that occur when multiple motion platforms traverse the same real-world scene through loop closure detection. Furthermore, it provides the limiting factor of loop closure, which can be used to optimize the system’s correlation back-end in order to achieve more accurate pose estimation and better global consistency mapping. The correlated post-detected candidate frames and the current frame detected in the first stage can be used as the global representation vector of the fine-matched image in the second stage after post-processing of the features. In addition, the similarity expression between the current frame image and any candidate frame image is shown in Eq (13).


$$Z(m,s)=\max(\frac{y_{m}^{T}}{{\left\|y_{m}\right\|}_{2}}\cdot \frac{y_{s}}{{\left\|y_{s}\right\|}_{2}},0)$$ (13)

In Eq (13), *Z*(*m*,*s*) denotes the similarity between the current frame image and any candidate frame image. *y*_*m*_ denotes the compressed global feature descriptor corresponding to the current frame image. *y*_*s*_ denotes the compressed global feature descriptor corresponding to the candidate frame image. ‖∙‖_2_ denotes the L2 paradigm, which is used to normalize the global representation descriptor of the image. Assuming that the relevant similarity metric value is greater than a set fixed threshold, a loop closure detection candidate frame is considered to be obtained. Ultimately, a geometric consistency test needs to be performed on the candidate frames after the corresponding loop closure is detected to determine whether the loop closure is valid or not. The study uses frame-map matching to geometrically verify the loop closures and to calculate the relative positional parameters between the current frame and the loop closure frame. In the back-end optimization of a multi-sensor fusion system, a factor graph under multi-sensors is analyzed. The factor graph consists of variable nodes representing the actual state of the system and four factor nodes representing the measurement constraints, including IMU pre-integration, LIDAR odometry, visual odometry, and loop closure factors. The four factors are used to construct the multi-sensor factor graph in the back-end module of the system and the optimal incremental smoothing method is applied to obtain the optimal values of the state estimation parameters.

To meet the real-time requirement of the system, a fixed length sliding window strategy is used for joint optimization, where the current frame and fixed length history frames are optimized in a sliding window. Moreover, when the system state frames within the sliding window exceeds a threshold, the history frames are truncated, after which a new sliding window is established. Meanwhile, in order to ensure sufficient quantitative constraints, 50 state nodes are reserved for the optimal sliding window of the factor graph under the premise of meeting real-time requirements. Optimization of the factor graph is carried out after the factor graph has been constructed, i.e., a set of optimal system state parameters are solved so that all edges of the factor graph are optimized. The optimal solution of the factor graph is derived as a nonlinear least square problem, and the corresponding expressions are shown in Eqs (14) and (15).


$$X=\underset{X}{\arg\;\min}\frac{1}{2}\{\begin{array}{l} \sum_{j\in \{0,\cdots n^{\prime }\}}\xi ({\left\|d_{L}({\mathbb{N}}_{L_{j}}^{W},X)\right\|}_{\sum L_{j}}^{2})+\sum_{j\in \{0,\cdots n^{\prime }\}}\xi ({\left\|d_{E}({\mathbb{N}}_{E_{j}}^{W},X)\right\|}_{\sum E_{j}}^{2})\\ +\sum_{j\in \{0,\cdots n^{\prime }\}}\xi ({\left\|d_{A}({\mathbb{N}}_{A_{j+1}}^{A_{j}},X)\right\|}_{\sum_{A_{j+1}}^{A_{j}}}^{2})+\sum_{j\in \{0,\cdots n^{\prime }\}}\xi ({\left\|d_{le}({\mathbb{N}}_{A_{le}}^{A_{j}},X)\right\|}_{\sum_{le}^{j}}^{2}) \end{array}\}$$ (14)

In Eq (14), *X* denotes the full set of system-related state variables that actually need to be estimated within the sliding window. $d_{L}({\mathbb{N}}_{L_{j}}^{W},X)$ denotes the LIDAR odometry factor. $d_{E}({\mathbb{N}}_{E_{j}}^{W},X)$ denotes the visual odometry factor. $d_{A}({\mathbb{N}}_{A_{j+1}}^{A_{j}},X)$ denotes the IMU pre-integration factor. $d_{le}({\mathbb{N}}_{A_{le}}^{A_{j}},X)$ denotes the loop closure factor. *ξ* denotes the Cauchy robust kernel function. Σ_(∙)_ is the covariance matrix.


$$X=\{x_{0},x_{1},\cdots ,x_{n^{\prime }}\}$$ (15)

**Fig 5:**
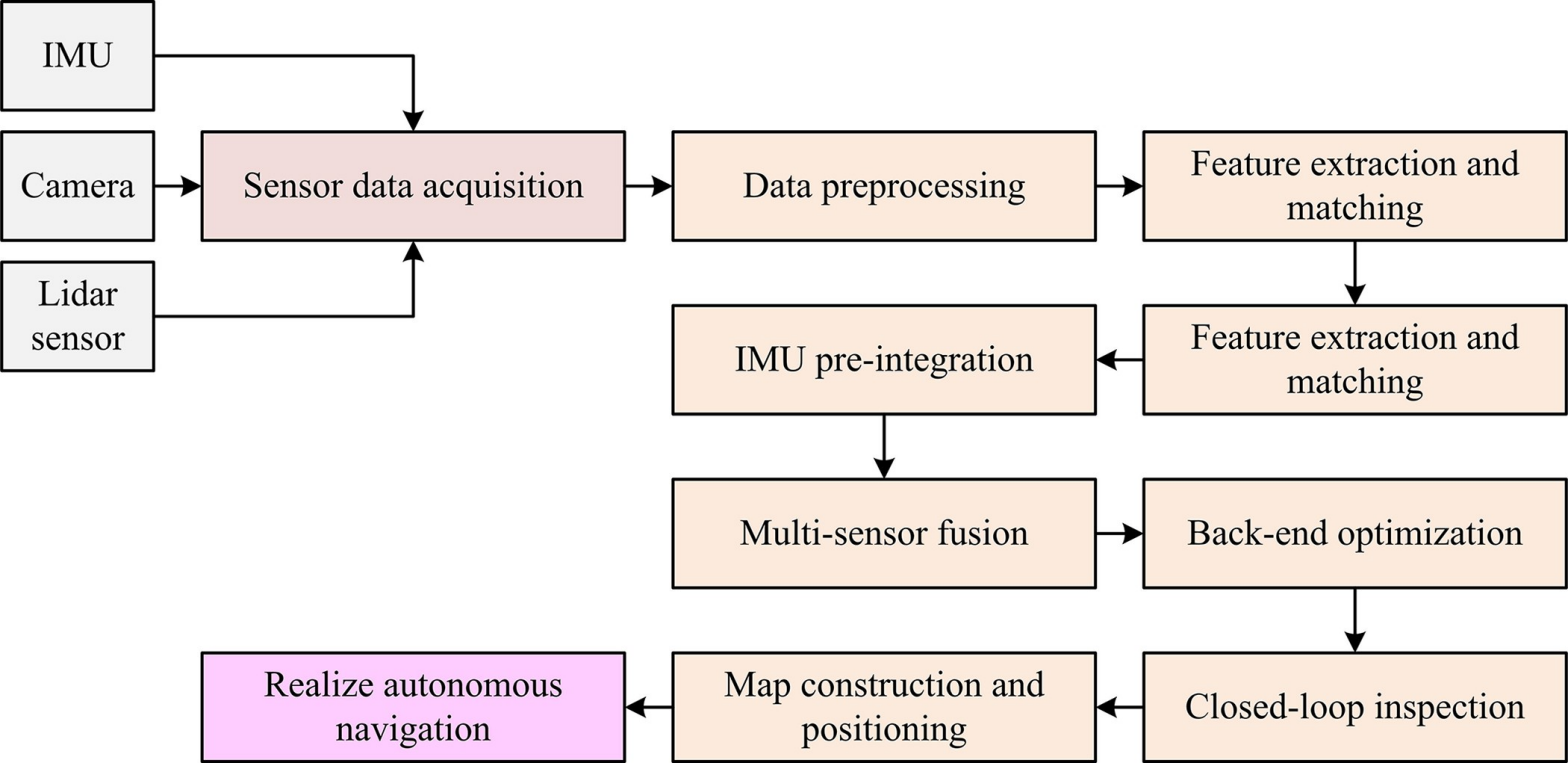
Multi-sensor fusion process and algorithm workflow.

In Eq (15), *n*′ denotes the length of the sliding window. Eqs (14) and (15) optimize the backend of factor graphs in multi-sensor fusion. On this basis, the conversion of the LiDAR point cloud according to the positional parameters is achieved through the output of the back-end optimization module and transformed into the data under the world coordinate system, so as to construct the global map. In terms of data pre-processing, the 3D-2D matching between the LiDAR point cloud in the camera’s field of view and the imagery is achieved through external parameters in the live reconstruction. This is achieved by synchronizing the LIDAR and the camera in time and space. The color or semantic information of the imagery is then mapped onto the point cloud to construct the global map containing the color texture or semantic information. In addition to this, in the process of RR, the state estimation method of moving objects can be used to exclude them from the global map to ensure that the constructed global map is not contaminated by moving objects. Finally, the multi-sensor fusion process and algorithm workflow proposed by the research are shown in Fig 5.

In Fig 5, the research collects data through IMU, camera and liDAR, and then performs pre-processing and time synchronization. Next, feature extraction and matching are used to correlate different sensor information, while IMU pre-integration techniques are used to estimate motion changes. Finally, by constructing a factor map and applying an optimization algorithm, state estimation and closed-loop detection are realized, so that accurate map construction and autonomous navigation can be performed in complex environments.

## 4. Experimental validation of RDAN-oriented multi-sensor localisation and RR methods

Experiments are conducted to evaluate the performance of the proposed multi-sensor fusion technique under a variety of real-world conditions. The experimental data are derived from the Urban Navigation (UrbanNav) dataset and the Multimodal Multi-Scene Ground Robot dataset (M2DGR), covering both urban environments and indoor scenes. The M2DGR dataset is a multi-sensor and multi-scenario SLAM dataset designed for ground robots. The M2DGR dataset comprises 36 sequences, totaling approximately 1TB of data, collected in various scenarios, both indoors and outdoors. The experiment is conducted on a hardware platform that is equipped with high-performance computing resources, ensuring the algorithm’s real-time operation. The RD is equipped with key sensors, including Lidar, cameras, and IMUs, to achieve high-precision environmental perception and autonomous navigation. The navigation system of the RD utilizes advanced data preprocessing, front-end odometers, and back-end optimization modules, all of which are meticulously designed and integrated to enhance the robot’s navigation ability in complex environments. This study corrects the motion distortion of lidar point cloud by utilizing IMU data, achieves time synchronization of different sensor data, and performs online calibration of the external parameters of LIdar and camera. Additionally, the data preprocessing process involves extracting essential features from the point cloud and applying denoising and filtering algorithms to enhance the input quality of subsequent data fusion and navigation algorithms.

In the experiments, open source SLAM algorithm is selected for comparative analysis. It consists of the LIDAR odometry and mapping (LOAM) algorithm, the lightweight and ground-optimized LIDAR odometry and mapping on variable terrain (LeGO-LOAM) [24,25]. Laser inertial navigation system (LINS), tightly coupled 3D LIO and mapping (LIO-Mapping), tightly coupled LIDAR-inertial odometry via smoothing and mapping (LIO-SAM) [26–28]. Simultaneous location and mapping 3 (ORB-SLAM3), monocular visual-inertial system (VINS-Mono), visual-inertial system fusion (VISO) for rapid and rotational orientation. Visual-inertial system fusion (VINS-Fusion), and direct LIDAR odometry (DLO) [29–31]. Simultaneously select three algorithms from literature that are closely related to the article for comparison, and set them as algorithms A to C. Algorithm A, proposed in reference [9], is an efficient and universal multi-sensor fusion method based on moving horizon estimation. Algorithm B, proposed in reference [12], is based on image sequence and wheel-inertia self-motion results. Algorithm C, proposed in reference [13], is a multi-sensor data fusion algorithm based on a nonlinear filter. The study selects these specific SLAM algorithms as benchmarks for comparative analysis because they represent the current advanced technology in the field of robot navigation and map construction. Each algorithm has unique characteristics, ranging from traditional lidar odometry to fusion methods that combine visual and inertial sensor data, as well as algorithms optimized for specific environments and application scenarios.

**Fig 6:**
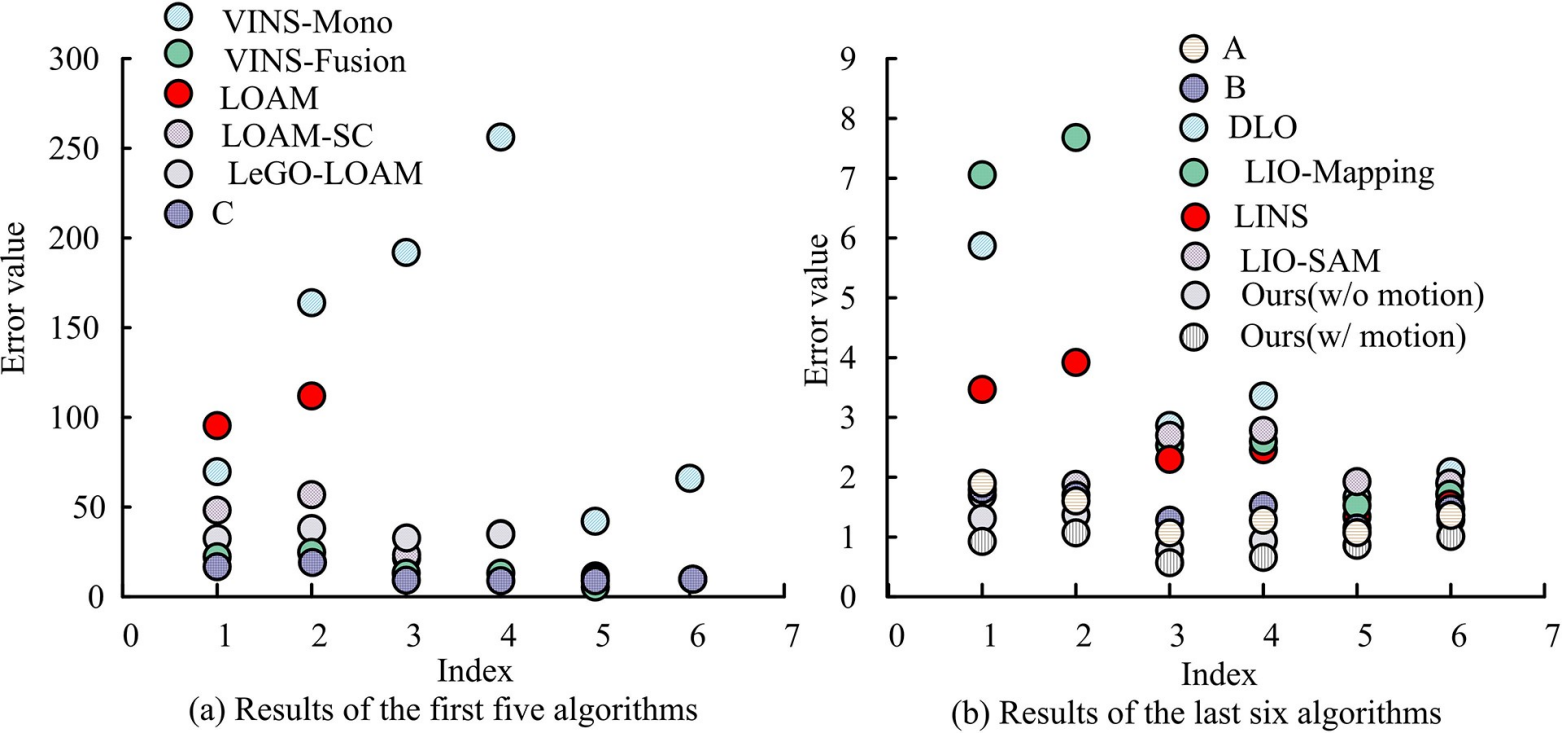
Positioning error results of different algorithms in the urban nav dataset.

An Intel system equipped with a central processor with a maximum main frequency of 3.6 GHz and a computer with 32 GB of RAM are selected as the testing hardware platform for the experimental environment. In the actual comparison, considering the uncertainty of the system under multi-threading and the related algorithm robustness, the experimental process is run five times for each test sequence and the mean value of the positioning error is taken as the final experimental result. Absolute trajectory error (ATE) and relative attitude error (RPE) are used as validation measures for positioning results in UrbanNav dataset. Under these two measures, root mean square error (RMSE) and mean square error (MMSE) are selected to evaluate the positioning accuracy of the algorithm. As a result, the localization error results of different algorithms in UrbanNav dataset are shown in Fig 6.

In Fig 6, horizontal axes 1 to 6 indicate the mean and RMSE of ATE, mean and RMSE of translation (%) under RPE, and mean and RMSE of rotation (°/100m). Comprehensively, Fig 6 shows that the RMSE value of the VINS-Mono algorithm is 163.97, which indicates that it suffers from a serious localization failure. the VINS-Fusion algorithm’s RMSE value is 24.83, which is at a lower level, and its RMSE value under PRE is 13.22% for translation and 8.51°/100 m. In addition, the ATE errors of LOAM, LOAM-SC, and LeGO-LOAM algorithms are much larger than those of VINS-Fusion algorithm. In addition, although the error values of Algorithm A, Algorithm B, and Algorithm C remain at a relatively low level, they are still relatively high compared to the research algorithms. In addition, the mean value under ATE of the proposed algorithm is 1.00 and RMSE is 1.07. the mean value under translation in RPE is 0.57% and RMSE is 0.66%, and the mean value under rotation is 0.91°/100m and RMSE is 1.01°/100m, which are lower than the comparison algorithms. In summary, the proposed algorithms have lower positioning errors, indicating that the RD is more adaptive to the environment in the role of the system under its auspices, thus enhancing the autonomous navigation capability. Eqs (14) and (15) optimize the back end of factor graphs in multi-sensor fusion.

**Fig 7:**
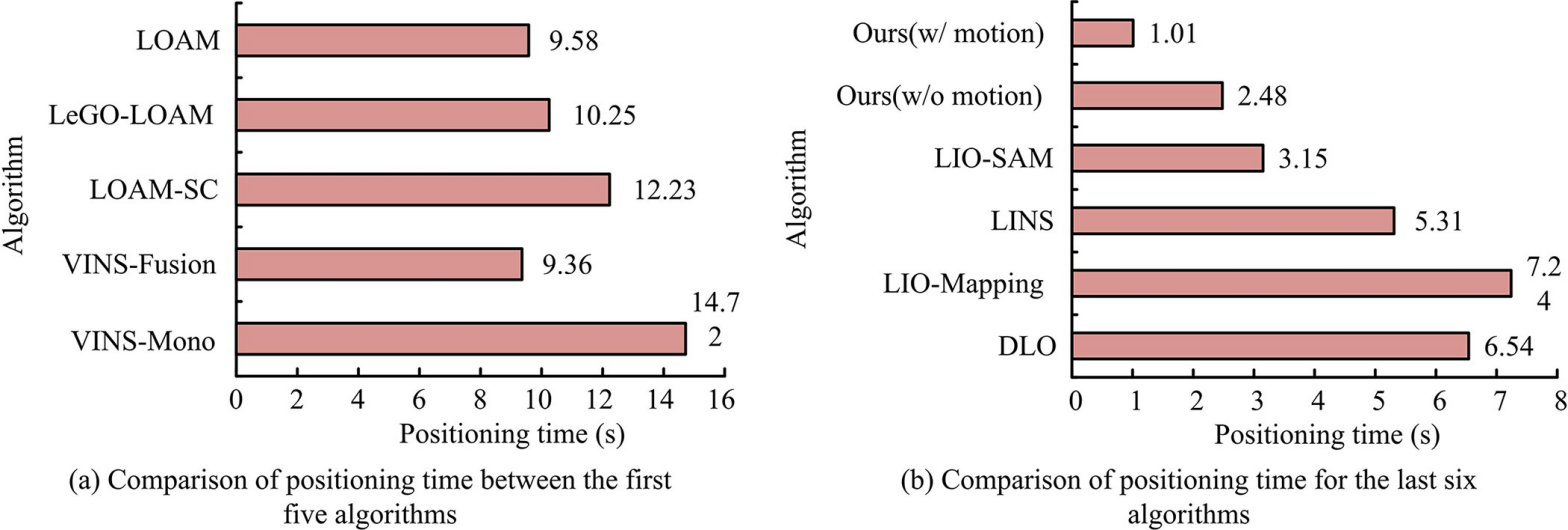
Comparison of different algorithm localization runtime.

**Fig 8:**
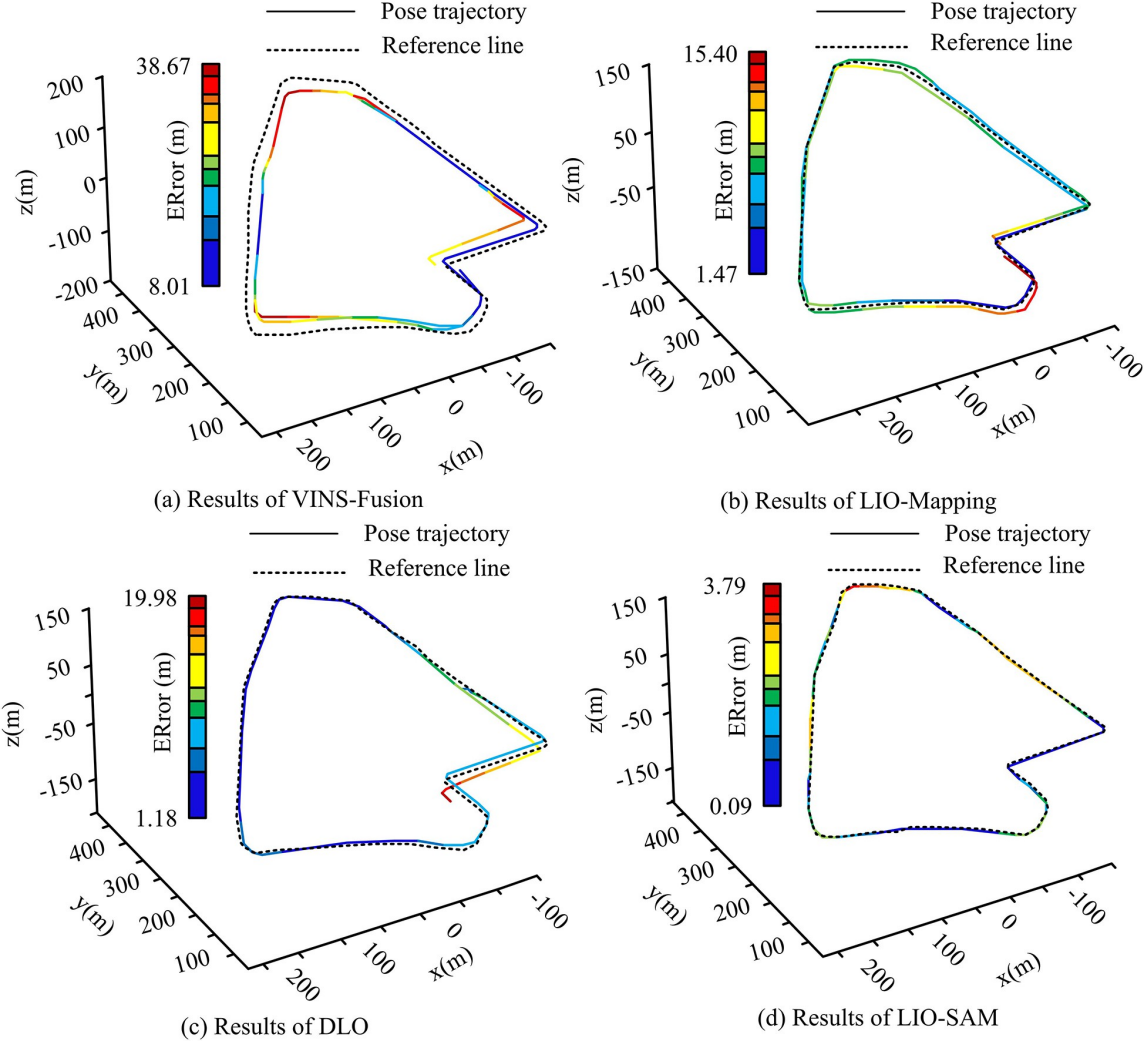
Comparison of position trajectories and truth values of different algorithms.

In Fig 7, the initial five algorithms had running times of 14.72 s, 9.36 s, 12.23 s, 10.25 s, and 9.558 s, respectively. Correspondingly, the latter six algorithms’ running times are 6.54 s, 7.21 s, 5.31 s, 3.15 s, 2.48 s, and 1.01 s, respectively. Overall, the research algorithm’s actual running time is significantly lower than the comparison, and it satisfactorily fulfills real-time requirements. Based on this, the study compares the actual positioning trajectories and the true values of the different algorithms to further validate the results. Among them, the baseline method in the figure refers to the method of Han K et al. [32]. Therefore, the analysis results of VINS-Fusion, LIO-Mapping, DIO, and LIO-SAM algorithms are shown in Fig 8.

**Fig 9:**
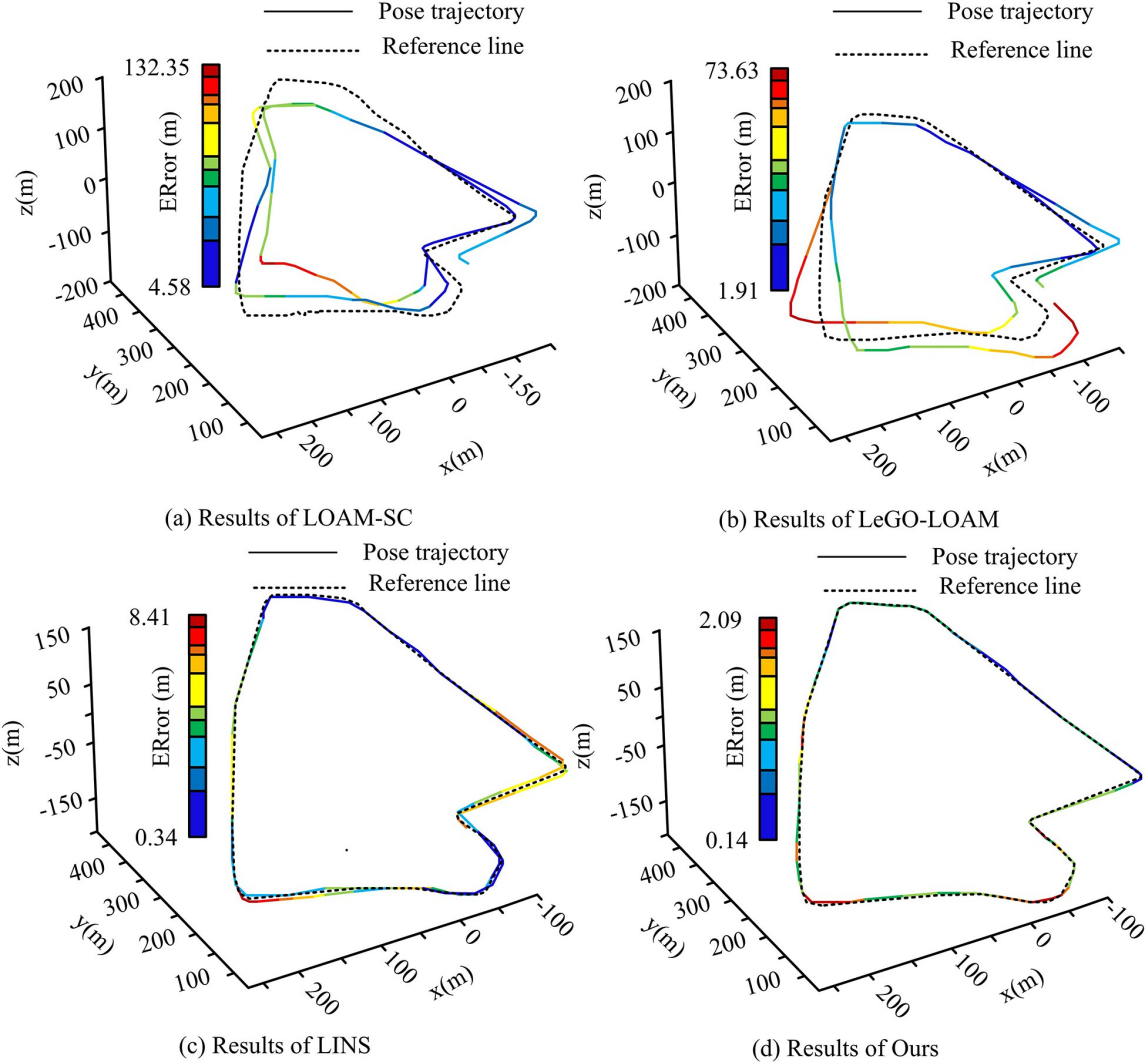
Comparison of position trajectories and truth values of different algorithms.

Fig 8 illustrates that the VINS-Fusion algorithm, due to its incorporation of a loop closure detection module, exhibits a considerable cumulative error. This is particularly evident in its front-end odometry, which generates a significant deviation in the actual position trajectory following loop closure detection and global optimization. This deviation is observed to deviate considerably from the true value of the trajectory. The LIO-Mapping, DIO, and LIO-SAM algorithms integrate LIDAR and IMU data fusion, resulting in improved positioning accuracy in complex real-world scenarios. Among the three, LIO-SAM has the lowest ATE error of 1.88 m. The analysis results of LOAM-SC, LeGO-LOAM, LINS, and the studied algorithms are shown in Fig 9.

Combined Fig 9 shows that the LOAM-SC algorithm exhibits the same results as the VINS-Fusion algorithm. the LINS algorithm exhibits the same results as the other three algorithms in Fig 8, but it has the lowest RPE errors, i.e., 2.46 per cent under translation and 1.90°/100 m under rotation. Figs 8 and 9 show that the research-proposed multi-sensor, multi-layer fusion localization method has the lowest actual localization error under the test scenario of the UrbanNav dataset. It indicates its effectiveness and an overall improvement of 42.85% in localization accuracy compared to the LIO-SAM algorithm. Meanwhile, the actual predicted positional trajectories of the research algorithm are likewise the closest to the true value, with the smallest deviation from the associated true value at different regional locations throughout the trajectory. In addition, in the M2DGR dataset, six sets of sequences are set as the actual test sequences for the outdoor scene data, and the specific parameters of each sequence are shown in Table 1.

**Table 1:** Specific parameters of the test sequence in the a multi-modal and multi-scenario dataset for ground robots dataset.

Sequence feature Sequence number	1	2	3	4	5	6
Sequence time	1228.00s	859.00s	495.00s	930s	908s	911s
Sequence length	1484.61m	840.42m	479.62m	1104.06m	916.79m	965.10m
Average speed	1.20m/s	0.99m/s	0.96m/s	1.20m/s	1.00m/s	1.05m/s
Description of sequence characteristics	Good daytime lighting, moving objects, and smooth movement	Intense changes in dark night light, loop back, and smooth movement	Intense changes in dark night light and smooth movement	Intense changes in dark night light, rapid movement, and intense exercise	Good daytime lighting, fast movement and vigorous exercise	Good daytime lighting, fast movement and vigorous exercise

**Fig 10:**
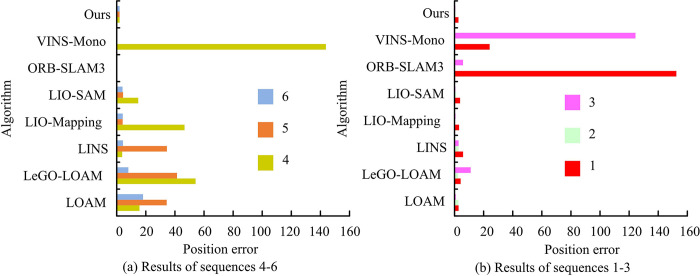
Positioning errors of different algorithms on the a multi-modal and multi-scenario dataset for ground robots dataset.

In Table 1, Sequences 1, 5 and 6 are acquired during the daytime, while the other three groups are at night, and the night is in a low-light scenario. Moreover, Sequence 2 incorporates a comprehensive loop closure to examine the efficiency of the loop closure detection algorithm and bolster the role of positioning accuracy. Sequence 4, Sequence 5, and Sequence 6 are collected in the zigzag curves under sudden movements such as sharp turns, sharp stops, accelerations, and decelerations of the RD, respectively. It can verify the accuracy and stability of the proposed RD localization and RR method under complex movements. Meanwhile, RMSE in ATE is used to evaluate the positioning accuracy of the algorithm and the results are shown in Fig 10.

**Fig 11:**
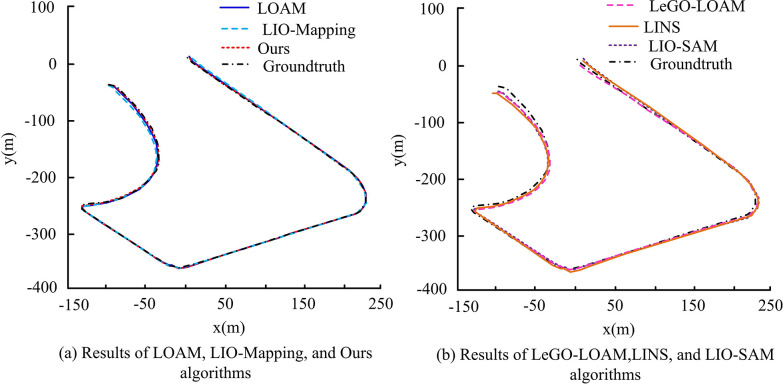
Positioning trajectories and pose truths of different simultaneous localization and mapping algorithms under Sequence 1.

Comprehensive Fig 10 shows that the visual SLAM algorithms ORB-SLAM3 and VINS-Mono have RMSE values of 152.45 and 24.15 in Sequence 1. VINS-Mono exhibits an RMSE value of 124.35 in Sequence 3 and 143.76 in Sequence 4, respectively, which are considerably higher than those of the comparison algorithms. Furthermore, it has failed to localize on sequences 2, 5, and 6, indicating a localization failure. The RMSE values of the research algorithm in the five sequences are 2.72, 0.80, 0.61, 1.97, 1.96, and 1.99. Overall, the research algorithm shows high performance under different complexities, which proves the effectiveness of the proposed multi-sensor multilevel localization and RR system in RDAN. The three day-time sequence data are extracted individually, in verifying the localization trajectory and positional truth of different SLAM algorithms under the three sequences. Among them, the results under Sequence 1 are shown in Fig 11.

**Fig 12:**
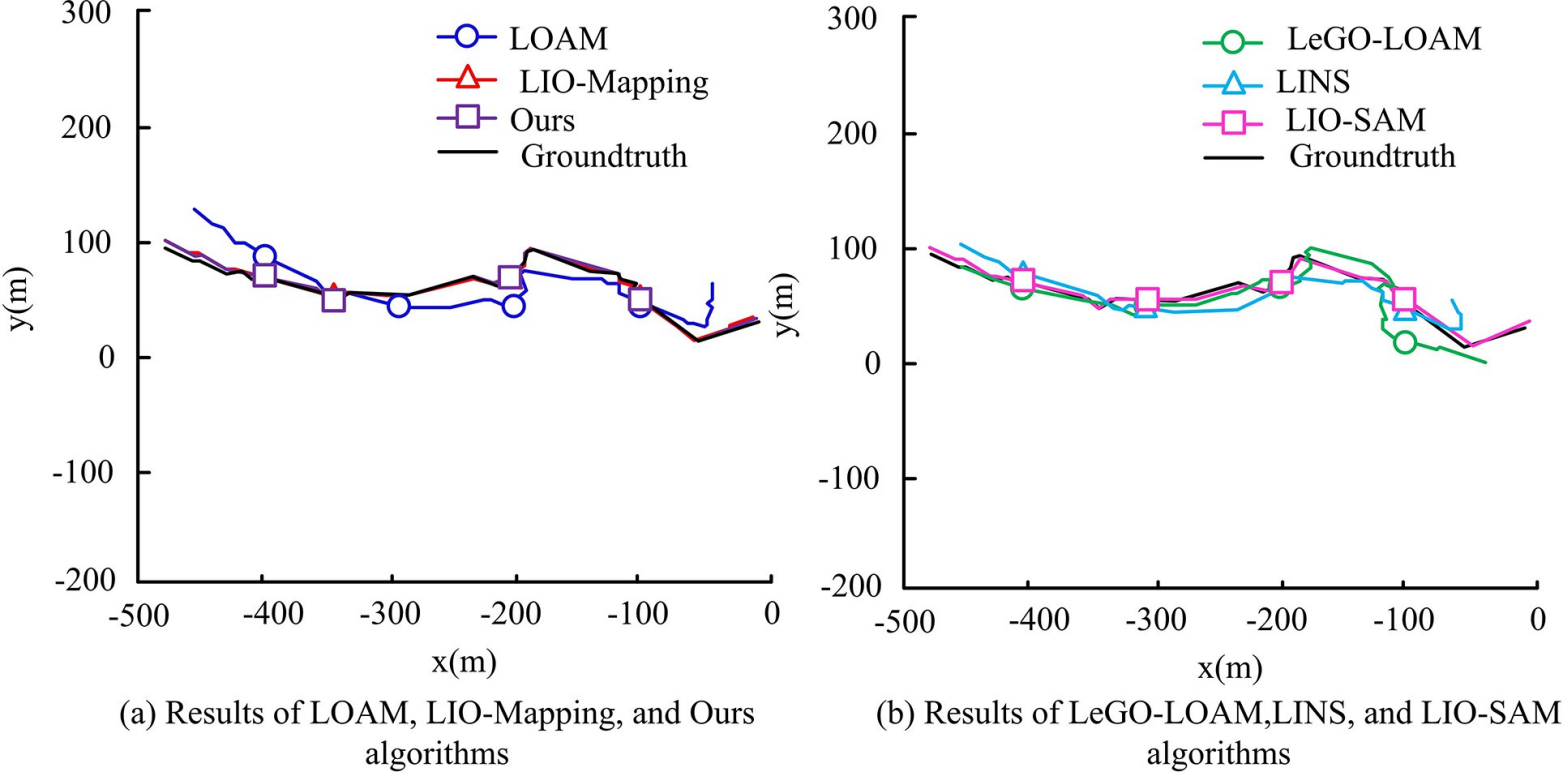
Positioning trajectories and pose truths of different simultaneous localization and mapping algorithms under Sequence 5.

In Fig 11, the positioning error across different algorithms in the sequence is relatively consistent, indicating no significant variance between the actual predicted trajectories. All algorithms approaches the true trajectory value more closely. Among them, the research proposes that the algorithm is closest to the true value of the trajectory, almost overlapping, and the comprehensive comparison is better. In addition, the results under Sequence 5 are shown in Fig 12.

**Fig 13:**
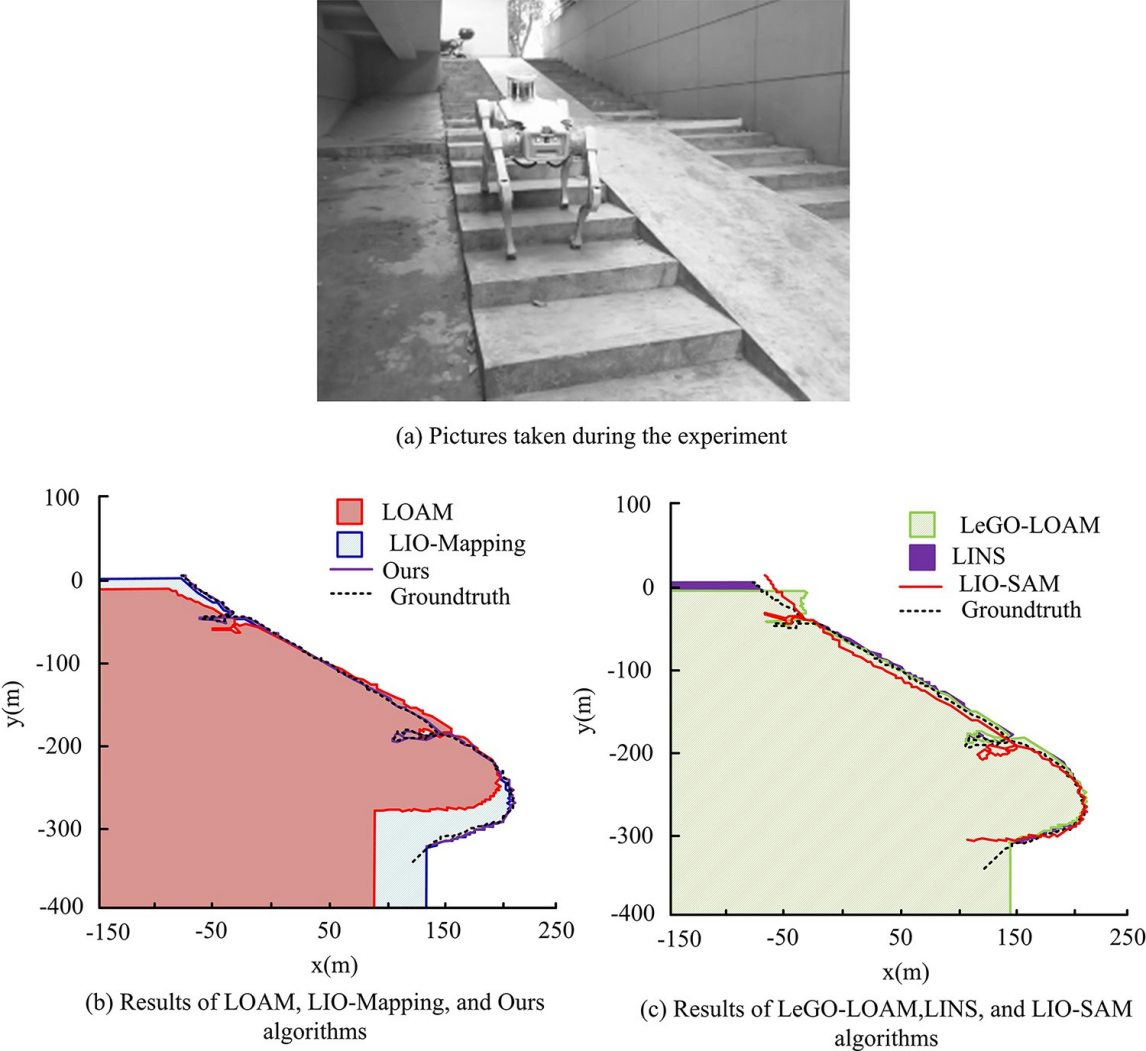
Positioning trajectories and pose truths of different simultaneous localization and mapping algorithms under Sequence 6.

In Fig 12, the comparison between the different algorithms shows significant differences in terms of changes in the horizontal axis, with some algorithms showing significant deviations. Among them, the LOAM, LeGO-LOAM, and LINS algorithms show significant localization errors, and the trajectories drift significantly with respect to the true values. Additionally, the LOAM and LeGO-LOAM algorithms solely rely on LIDAR point cloud data as inputs. However, when the RD increases, it results in considerable motion distortion, leading to significant deviations in position estimation. This distortion can adversely affect navigation when no auxiliary sensors are available to correct for it. The research algorithm likewise almost overlaps with the true value and gives the best results. The results under Sequence 6 are shown in Fig 13.

In Fig 13, the proposed method fuses multi-sensors at multiple levels in each module of the system, resulting in higher positioning accuracy than LIO-Mapping and LIO-SAM, and the accuracy and stability of scanning matching are significantly improved. To verify the practicality of the proposed approach, the study implements it in real RD situations and places it in actual scenarios, creating two experimental scenarios: the science and technology innovation building laboratory (TIB-LAB) and the long corridor of the science and technology innovation building (TIB-Corridor). TIB-LAB and TIB-Corridor both which contain indoor and global position system (GPS). Therefore, the comparison results of different algorithms for positioning in the two scenarios are shown in Table 2.

**Table 2:** Comparison of positioning results between different algorithms in two scenarios.

TIB indoor dataset				
Method Scenario	Indoor lab (Translation) (m)	Indoor lab (Rotate) (°)	Indoor corridor (Translation) (m)	Indoor corridor (Rotate) (°)
LOAM	0.28	5.07	8.38	6.40
LEGO-LOAM	0.29	6.05	24.24	18.09
DLO	0.14	5.95	16.11	8.10
LIO-Mapping	-	-	0.23	5.36
LIO-SAM (w/o loop)	0.10	4.57	4.46	3.90
LIO-SAM (w/loop)	0.07	4.44	0.07	3.58
The proposed method (w/o loop)	0.14	4.29	0.07	2.28
The proposed method (w/loop)	0.04	4.34	0.02	1.86
TIB-GPS Datasets				
Method Index	RMSE (m)		Mean (m)	
LOAM	14.57		11.19	
LEGO-LOAM	1.30		1.24	
DLO	1.67		1.46	
LIO-Mapping	1.01		0.99	
LIO-SAM (w/o loop)	1.06		1.03	
LIO-SAM (w/loop)	0.94		0.93	
The proposed method (w/o loop)	0.89		0.90	

**Fig 14:**
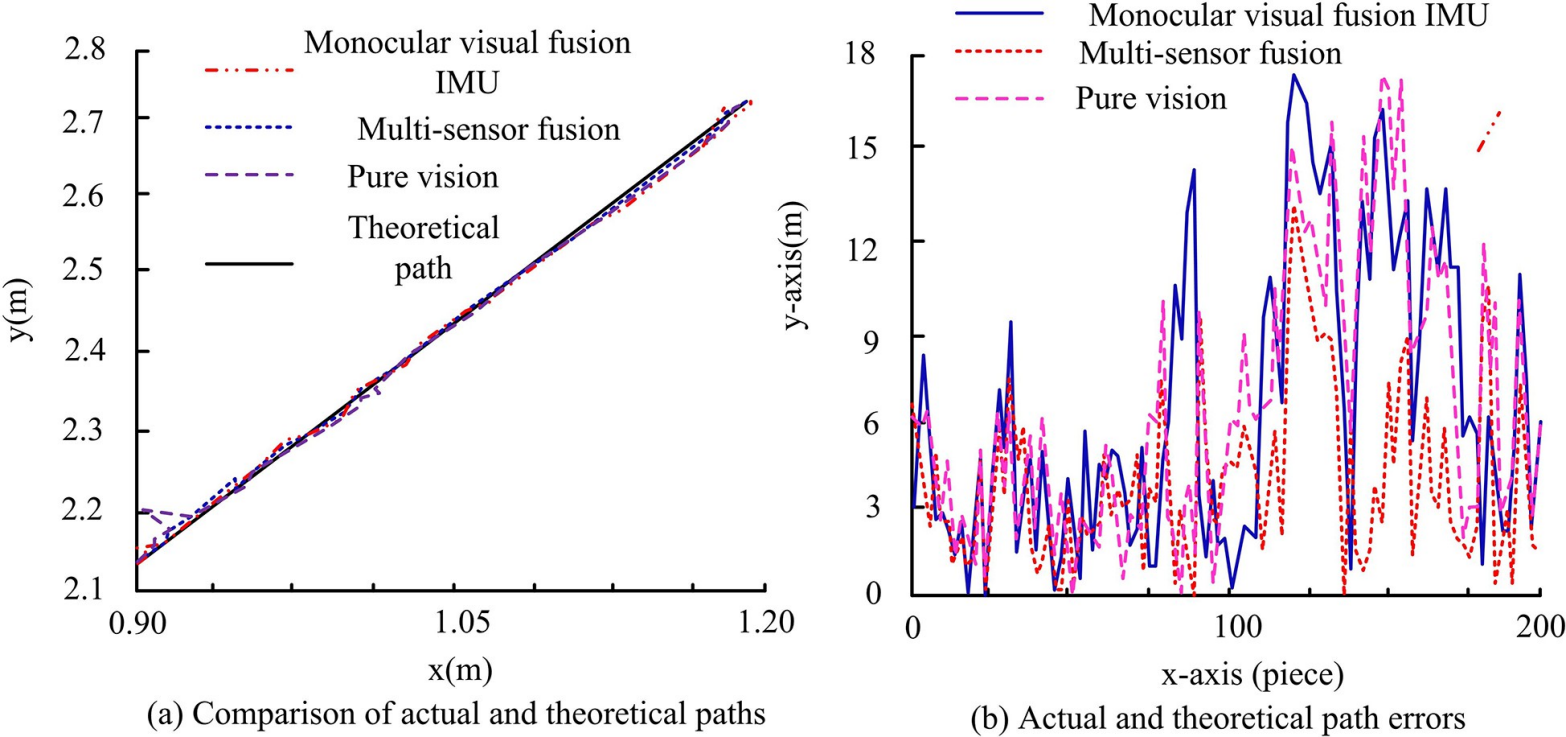
Comparison results of error between indoor running path and theoretical path of the robot dog.

In Table 2, the error of the relevant values is the average error. Table 2 illustrates the results of the TIB-indoor dataset during the laboratory indoor test sequence. It is evident that for other methods, the values range from 0.08m to 0.29m under translation error. Notably, LIO-Mapping initialization failed, possibly due to insufficient motion excitation. On the other hand, the research algorithm records a value of 0.04m, which is significantly lower than the compared methods. When comparing rotation errors, the other algorithms produced values of 5.07°, 6.05°, 5.95°, 4.57°, 4.44°, and 4.29°. Meanwhile, the research algorithm has a slightly higher value in comparison to calculations without the research algorithm. However, it is significantly lower than the values yielded by other algorithms. Under the translation error in the indoor long corridor test sequence, the research algorithm has a value of 0.02m and a rotation error of 1.86°, both lower than the comparison algorithm. In addition, in the dataset TIB-GPS, the positioning error RMSE is 0.89 and the mean value is 0.90, again lower than the comparison algorithms. Taken together, the research algorithm possesses validity in practical applications, i.e., the multi-sensor multilayer fusion localization method possesses practicality. Based on this, in order to verify the application of the proposed multi-sensor localization and RR method in the actual autonomous navigation of RD, three sets of data, i.e., single-sensor fusion IMU, pure vision, and the research method, are compared in the actual scenario. The results of which are shown in Fig 14 for the comparison of the RD indoor running path with the theoretical path error.

**Fig 15:**
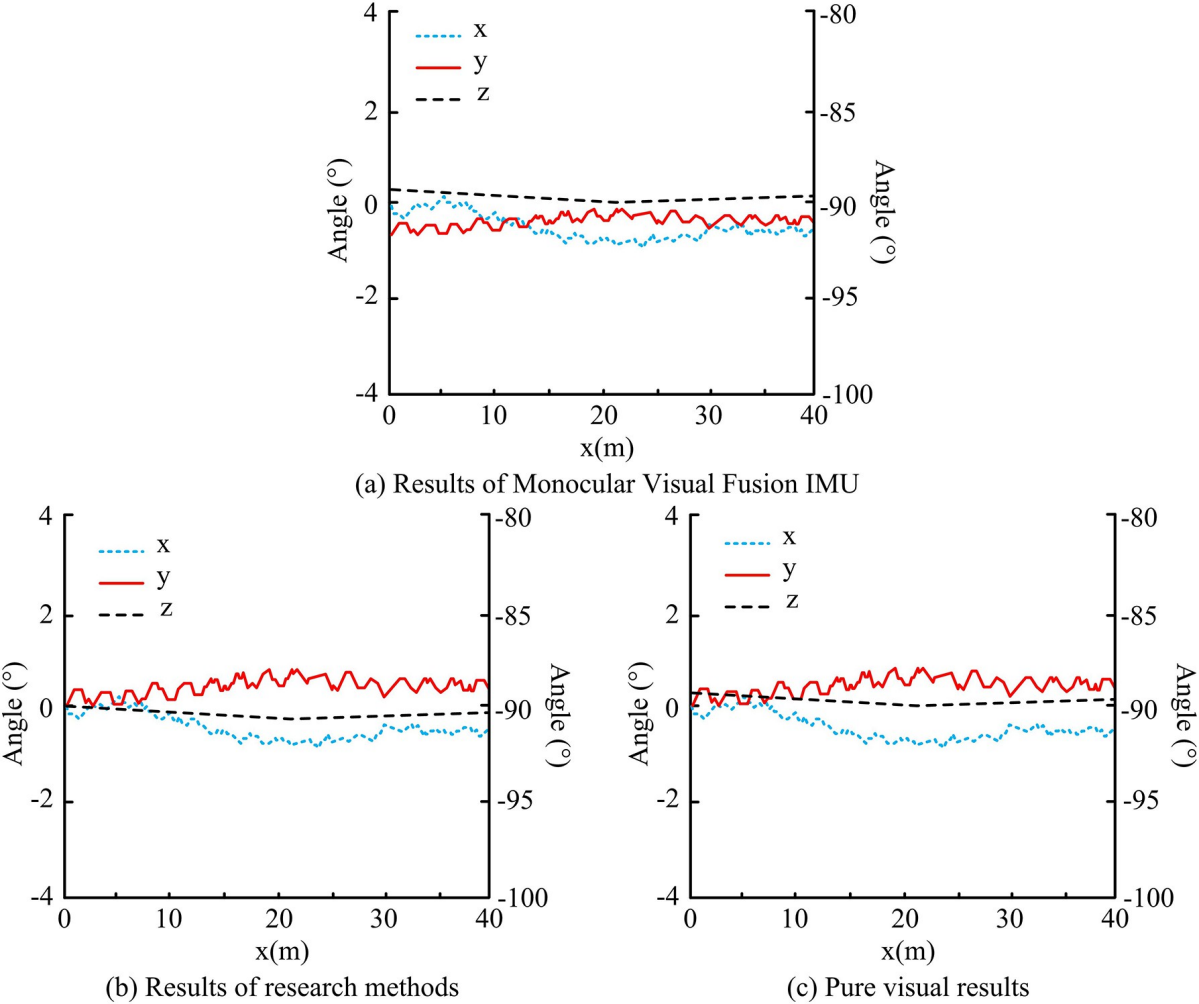
The result of the deviation of the angle axis between the RD and the three-dimensional point cloud coordinates during the actual movement process.

Comprehensive Fig 14 shows that the research method is closest to the theoretical path line, and the error between it and the theoretical path is the lowest in most cases, which is not more than 0.12m. Theoretical superiority of the research methodology is demonstrated by the results. In the context of practical RD, information is extracted in three distinct ways using the robot operating system (ROS). Based on this, the result of the size of the offset of the angle axis from the 3D point cloud coordinates during the actual movement of the RD is shown in Fig 15.

Comprehensive Fig 15 shows that the movement path offset of RD p-axis and q-axis under monocular vision and IMU fusion is maintained between -2° to 0°, while z-axis is maintained above -90° variation. Both p-axis and q-axis of the proposed multi-sensor fusion method of the study start at 0° to maintain the variation between -2° to 2°, while the z-axis maintains the variation at -90°. Due to the large amount of actual data, 200 sets of data are selected to be analyzed on the basis of Figs 14 and 15, and the obtained results of the theoretical and actual path errors of RD are shown in Table 3.

**Table 3:** Comparison results of RD theory and actual path errors.

Method index	Maximum error	Minimum error	Average error	Standard error	Maximum angle rotation	Minimum angle rotation	Average rotation
Monocular visual fusion IMU	15.579cm	0.068cm	6.549cm	3.949cm	0.046°	3.926e-06°	0.015°
Multi-sensor fusion	13.149cm	0.048cm	4.069cm	1.679cm	0.028°	3.678e-06°	0.008°
Pure vision	15.619cm	0.218cm	6.609cm	4.499cm	0.048°	4.934e-06°	0.024°

**Fig 16:**
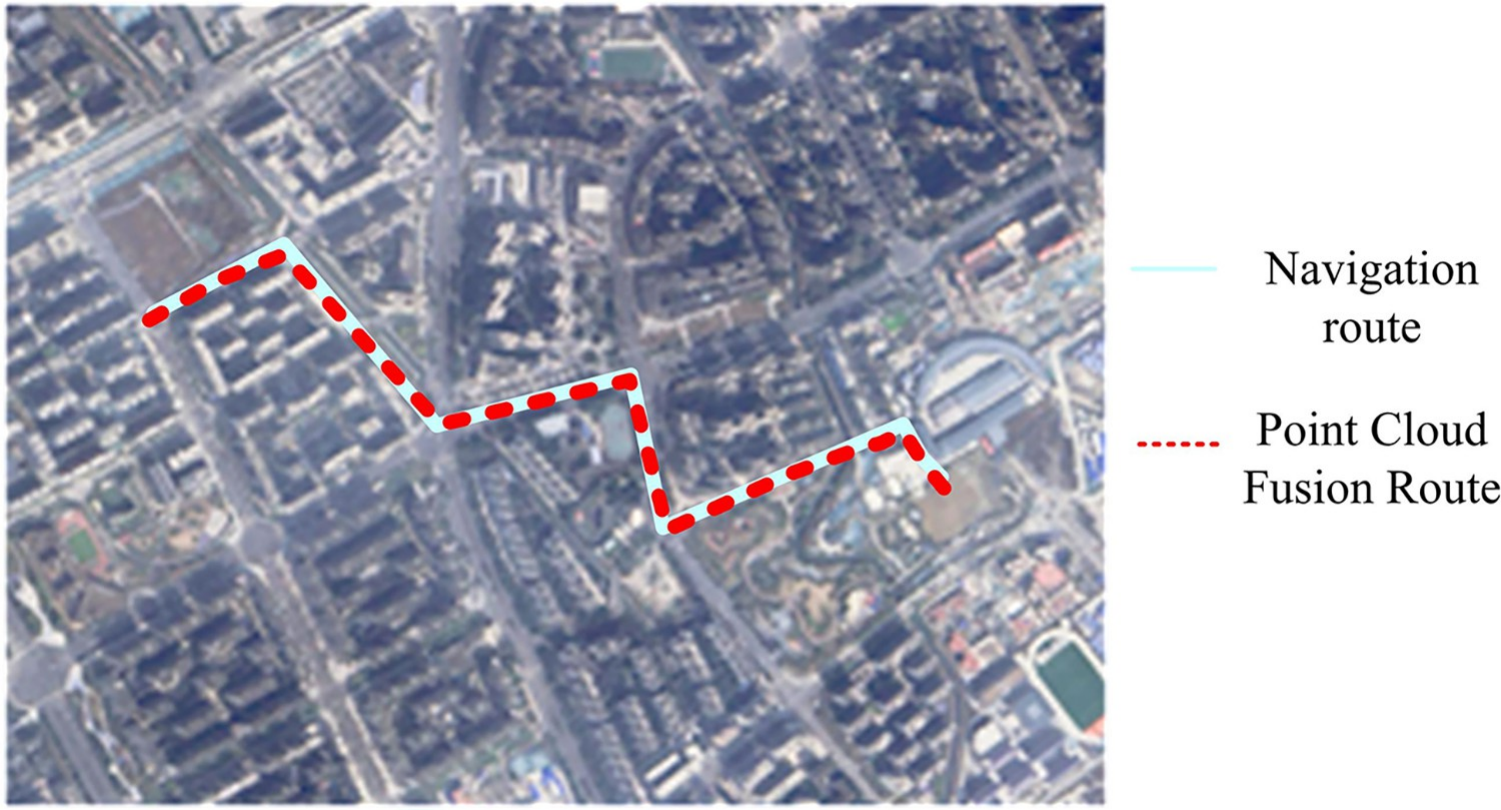
Point cloud fusion instance effect.

In Table 3, the maximum error of the multi-sensor fusion method proposed by the study is 13.149cm, the minimum error is 0.048cm, the average error value is 4.069cm, and the standard error value is 1.679cm. While the maximum angular rotation is 0.028 degrees, the minimum angular rotation is 3.678e-06 degrees, and the average rotation is 0.008 degrees, which are lower than the comparison method. In summary, the proposed method of the study has the smallest error, proving the superiority of the method in RD positioning and navigation in practical applications. To test the effectiveness of the research method’s point cloud instance fusion, it is implemented for navigation on a central road in a specific region. Fig 16 exhibits the obtained results.

Fig 16 is situated on a small urban street with complex transportation infrastructure, which includes obstacles such as small cars, electric vehicles, debris, and pedestrian flow. This complexity makes it suitable for navigation verification needs in practical research applications. Fig 16 indicates that the navigation and positioning results obtained from the fusion of point cloud data display that the overall positioning trajectory is consistent with the planned route. However, at the turning point, the route planning algorithm of Baidu Map is not optimized, leading to a deviation from the actual trajectory. Nonetheless, the fusion outcomes accurately reflect the driving position of the prototype RD. A new Dataset SubT-MRS Dataset is selected again for a new experiment. The dataset primarily captures RD motion data in complex underground environments and irregular terrain, covering multi-degree of freedom motion modes, including pitching, rolling, and swinging. Three sets of data from the SubT-MRS Dataset are selected, including covering complex terrain, dynamic obstacles and low light environment respectively. The experimental platform is a RD, and the sensors are configured as liDAR, IMU and RGB cameras. To ensure the fairness of the experiment, each set of experiments is run five times. Moreover, the ATE, path smoothness (RPE), and processing time (runtime) are recorded. In the experiment, the proposed multi-sensor fusion localization algorithm is employed for comparison with three mainstream SLAM algorithms (LOAM, LIO-SAM, ORB-SLAM3). The results are shown in Table 4.

**Table 4:** Experimental results in the SubT-MRS dataset.

Sequence number	Algorithm	Average trajectory error ATE (m)	Path smoothness RPE (%)	Runtime (s)
Sequence 1	LOAM	0.25	5.32	18.76
	LIO-SAM	0.21	4.89	17.25
	ORB-SLAM3	0.31	6.10	20.56
	Proposed method	0.12	3.85	15.02
Sequence 2	LOAM	0.29	6.01	19.33
	LIO-SAM	0.24	5.62	18.04
	ORB-SLAM3	0.36	6.54	21.44
	Proposed method	0.14	4.27	16.72
Sequence 3	LOAM	0.32	6.43	20.10
	LIO-SAM	0.28	5.98	19.03
	ORB-SLAM3	0.38	6.81	22.66
	Proposed method	0.16	4.62	17.88

As illustrated in Table 4, the ATE of the proposed algorithm is less than that of the other algorithms in all three groups of experiments. In Sequence 1 (complex terrain), the positioning error is 0.12 m, which is a significant improvement over LOAM (0.25 m) and LIO-SAM (0.21 m). For path smoothness, the RPE values also show the advantages of the proposed algorithm. In Sequence 2 (dynamic obstacle), the RPE of the proposed algorithm is 4.27% compared to 6.54% of ORB-SLAM3, and the smoothness is significantly improved. As for the running time, the running time of the proposed algorithm is lower than other algorithms in all experiments, especially in Sequence 3. Among them, the processing time is 17.88 s, while the processing time of ORB-SLAM3 reaches 22.66 s. This shows that the proposed algorithm has good real-time performance while maintaining high positioning accuracy.

## 5. Conclusion

The study suggested a multi-sensor localization and RR approach based on the use of 3D-LPCRF and validates its efficacy in order to solve the issue of degradation of localization and RR accuracy in mobile RDAN in complex real-world scenarios. The experiment’s outcomes showed that the average translation mean value and corresponding RMSE were 0.57% and 0.66%, respectively, in the RPE of the algorithm utilized in the proposed approach for the UrbanNav data set. Additionally, the mean value of rotation was 0.91°/100m with an RMSE of 1.01°/100m, both of which were smaller than those of the comparison algorithm and closer to the true value line. For the five sequences in the M2DGR data set, the research algorithm’s RMSE values were 2.72, 0.80, 0.61, 1.97, 1.96, and 1.99, which were lower than those of the comparison method. It showed that the path lines are considerably more closely related to the true value lines. The research algorithm’s value of 0.04m was significantly lower than the comparison algorithm’s value under translation error in laboratory indoor test sequences. In most cases, it had the lowest error with theoretical paths, not exceeding 0.12m when applied to real scenarios. In contrast to the comparative approaches, the highest inaccuracy following its application in RD was 13.149 cm, and the average rotation was 0.008 degrees. The suggested multi-sensor localization and RR approach, when combined, had a high performance for improving RD localization and navigation and was also successful in real-world settings. In-depth assessments will be required in the future because the data used by the suggested approach of fusing three sensors is not rich enough and more complex scenarios have not been confirmed. The suggested approach for multi-sensor multi-level fusion positioning and scene reconstruction relies solely on LIDAR, camera, and IMU sensors. Future research will examine the integration of additional sensor types like wheel odometrys, global satellite positioning systems, event cameras, millimeter wave LiDAR, etc. This will enhance the precision and stability of positioning and scene reconstruction in complex dynamic scenarios involving multi-sensor systems.

## 6. Discussion

Compared to traditional automated tasks, unmanned tasks had the advantage of obtaining higher autonomy through intelligent algorithms, thereby accurately completing more complex tasks and further achieving the ultimate goal of "machine replacing human". Therefore, a multi-sensor localization and scene reconstruction method was proposed based on the fusion of 3D LIDAR point clouds, and its effectiveness was verified.

The experimental results showed that the mean value and RMSE value of the proposed algorithm ATE were 1.00 and 1.07 respectively. The mean value under translation in RPE was 0.57%, RMSE was 0.66%, mean value under rotation was 0.91°/100m, and RMSE value was 1.01°/100m, both of which were lower than the comparison algorithm. This result was also superior to the method proposed by Bai X et al. [33]. The actual running time of the research algorithm was much lower than that of the comparison, which basically met the real-time requirements. In addition, the RMSE values of the research algorithm in five sequences were 2.72, 0.80, 0.61, 1.97, 1.96, and 1.99, respectively. It indicated that it exhibited high performance in different complex situations. The maximum error of the proposed multi-sensor fusion method was 13.149cm, the minimum error was 0.048cm, the average error value was 4.069cm, and the standard error value was 1.679cm. The maximum angular rotation was 0.028 degrees, the minimum angular rotation was 3.678e-06 degrees, and the average rotation was 0.008 degrees. Both of which are lower than the comparison method. This result was also significantly superior to the method proposed by Morales J et al. [34].

The research has improved the positioning accuracy and robustness of the RD in complex environments by using advanced multi-sensor fusion technology. This is achieved by combining three-dimensional LiDAR point clouds and IMU data. This fusion strategy enhances adaptability to dynamic environmental changes and ensures real-time system performance. As a result, the RD can respond quickly and make accurate navigation decisions. Additionally, the study’s proposed method demonstrates strong multi-modal perception capabilities and system scalability. This provides a reliable technical foundation for autonomous navigation of RD in real-world scenarios and opens up possibilities for future applications in various fields, including search and rescue, environmental monitoring, and home services.

This research has made significant progress in the field of autonomous navigation of RD. However, there are still limitations, such as the computational burden of the algorithm and environmental adaptability. Deep learning and reinforcement learning can enhance the system’s perception and decision-making capabilities. At the same time, follow-up research should focus on the development of adaptive and online learning mechanisms that enable systems to dynamically adjust behavior and learn from experience.

## Supporting information

S1 Data (DOC)
