# Supplementary material for: Application of 3D point cloud and visual-inertial data fusion in Robot dog autonomous navigation
Source: PLoS One. 2025 Feb 11;20(2):e0317371. doi: 10.1371/journal.pone.0317371 (PMC11813145; doi:10.1371/journal.pone.0317371)
Supplement: S1 Data — (DOC) [file pone.0317371.s001.doc]

**The data in Figure 6. Positioning error results of different algorithms in the urban nav dataset**

|  | 1 | 2 | 3 | 4 | 5 | 6 |
| --- | --- | --- | --- | --- | --- | --- |
| VINS-Mono | 69.46 | 163.97 | 184.16% | 261.41% | 46.18°/100 m | 62.37°/100 m |
| VINS-Fusion | 16.51 | 24.83 | 10.19% | 13.22% | 17.13°/100 m | 8.51°/100 m |
| LOAM | 96.61 | 106.79 | 13.51% | 11.45% | 6.14°/100 m | 6.16°/100 m |
| LOAM-SC | 51.33 | 58.16 | 26.34% | 6.19% | 5.18°/100 m | 6.28°/100 m |
| LeGO-LOAM | 37.13 | 43.96 | 21.82% | 7.13% | 3.71°/100 m | 5.17°/100 m |
| C | 9.64 | 12.28 | 9.46% | 5.28% | 8.45°/100 m | 4.39°/100 m |
| A | 1.99 | 1.67 | 1.06% | 1.51% | 1.94°/100 m | 1.48°/100 m |
| B | 1.76 | 1.71 | 1.32% | 1.72% | 1.82°/100 m | 1.34°/100 m |
| DLO | 6.01 | 1.53 | 2.98% | 3.26% | 3.29°/100 m | 1.39°/100 m |
| LIO-Mapping | 7.14 | 7.86 | 2.61% | 2.67% | 1.89°/100 m | 1.64°/100 m |
| LINS | 3.51 | 1.72 | 2.32% | 2.56% | 1.36°/100 m | 1.71°/100 m |
| LIO-SAM | 1.69 | 1.54 | 1.03% | 1.46% | 1.28°/100 m | 1.77°/100 m |
| Ours(w/o motion) | 1.31 | 1.26 | 0.68% | 0.83% | 1.19°/100 m | 1.69°/100 m |
| Ours(w/ motion) | 1.00 | 1.07 | 0.57% | 0.66% | 0.91°/100m | 1.01°/100m |

**The data in Figure 7. Comparison of different algorithm localization runtime**

| Algorithm | Positioning time (s) |
| --- | --- |
| VINS-Mono | 14.27 |
| VINS-Fusion | 9.36 |
| LOAM | 12.23 |
| LOAM-SC | 10.25 |
| LeGO-LOAM | 9.58 |
| DLO | 6.54 |
| LIO-Mapping | 7.24 |
| LINS | 5.31 |
| LIO-SAM | 3.15 |
| Ours(w/o motion) | 2.48 |
| Ours(w/ motion) | 1.01 |

**The data in Figure 8. Comparison of position trajectories and truth values of different algorithms**

| Real coordinate | VINS-Fusion | LIO-Mapping | DLO | LIO-SAM |
| --- | --- | --- | --- | --- |
| (0,100,0) | (0,117,0) | (0,94,0) | (0,101,0) | (0,99,0) |
| (100,200,0) | (104,245,0) | (101,202,0) | (99,202,0) | (103,202,0) |
| (150,230,0) | (134,230,0) | (148,231,0) | (151,229,0) | (150,229,0) |
| (100,300,0) | (106,279,0) | (100,306,0) | (104,301,0) | (98,300,0) |
| (0,400,0) | (0,382,0) | (0,400,0) | (3,410,0) | (2,401,0) |
| (-100,350,0) | (-96,341,0) | (-106,348,0) | (-106,361,0) | (-101,350,0) |
| (-150,200,0) | (-146,178,0) | (-144,202,0) | (-153,204,0) | (-151,203,0) |
| (-100,100,0) | (-103,98,0) | (-104,113,0) | (-100,134,0) | (-100,102,0) |

**The data in Figure 9. Comparison of position trajectories and truth values of different algorithms**

| Real coordinate | LOAM-SC | LeGO-LOAM | LINS | Ours |
| --- | --- | --- | --- | --- |
| (0,100,0) | (0,146,0) | (-26,16,0) | (6,102,0) | (0,101,0) |
| (100,200,0) | (100,264,0) | (82,190,0) | (98,203,0) | (100,199,0) |
| (150,230,0) | (150,298,0) | (150,261,0) | (151,230,0) | (150,230,0) |
| (100,300,0) | (121,336,0) | (88,316,0) | (101,300,0) | (100,301,0) |
| (0,400,0) | (32,376,0) | (3,410,0) | (0,405,0) | (0,401,0) |
| (-200,350,0) | (-194,318,0) | (-188,334,0) | (-99,353,0) | (-100,350,0) |
| (-150,200,0) | (-131,246,0) | (-150,196,0) | (-151,200,0) | (-150,200,0) |
| (-200,200,0) | (-164,168,0) | (-166,132,0) | (-102,104,0) | (-101,100,0) |

**The data in Figure 10. Positioning errors of different algorithms on the a multi-modal and multi-scenario dataset for ground robots dataset**

| - | Sequences1 | Sequences2 | Sequences3 | Sequences4 | Sequences5 | Sequences6 |
| --- | --- | --- | --- | --- | --- | --- |
| LOAM | 2.82 | 2.69 | 0.91 | 15.78 | 34.36 | 18.49 |
| LeGO-LOAM | 4.23 | 2.46 | 11.11 | 54.22 | 41.45 | 8.06 |
| LINS | 5.84 | 2.85 | 2.82 | 3.77 | 34.49 | 4.31 |
| LIO-Mapping | 3.26 | - | 0.73 | 46.61 | 4.11 | 4.19 |
| LIO-SAM | 3.86 | 1.14 | 0.66 | 14.81 | 4.39 | 4.04 |
| ORB-SLAM3 | 152.45 | - | 5.85 | - | - | - |
| VINS-Mono | 24.15 | - | 124.35 | 143.76 | - | - |
| Ours | 2.72 | 0.80 | 0.61 | 1.97 | 1.96 | 1.99 |

**The data in Figure 11. Positioning trajectories and pose truths of different simultaneous localization and mapping algorithms under Sequence 1**

| Real coordinate | LOAM | LIO-Mapping | Ours | LeGO-LOAM | LINS | LIO-SAM |
| --- | --- | --- | --- | --- | --- | --- |
| (-140,-250) | (-141,-252) | (-138,-254) | (-141,-250) | (-144,-252) | (-144,-252) | (-144,-248) |
| (-100,50) | (-103,52) | (-102,56) | (-100,50) | (-104,51) | (-102,51) | (-104,52) |
| (-10,-360) | (-8,-362) | (-8,-362) | (-10,-361) | (-11,-357) | (-8,-363) | (-7,-363) |
| (0,0) | (0,0) | (1,1) | (0,0) | (3,2) | (-4,2) | (-6,3) |
| (175,-270) | (174-276) | (171,-277) | (175,-270) | (174,-276) | (177,-273) | (174,-266) |

**The data in Figure 12. Positioning trajectories and pose truths of different simultaneous localization and mapping algorithms under Sequence 5**

| Real coordinate | LOAM | LIO-Mapping | Ours | LeGO-LOAM | LINS | LIO-SAM |
| --- | --- | --- | --- | --- | --- | --- |
| (-400,80) | (-400,91) | (-400,83) | (-400,81) | (-400,76) | (-400,88) | (-400,83) |
| (-300,60) | (-300,49) | (-300,56) | (-300,52) | (-300,49) | (-300,56) | (-300,54) |
| (-200,90) | (-200,50) | (-200,92) | (-200,89) | (-200,84) | (-200,93) | (-200,92) |
| (-100,50) | (-100,56) | (-100,56) | (-100,50) | (-100,16) | (-100,43) | (-100,58) |
| (0,20) | - | (0,22) | (0,20) | - | (0,21) | (0,22) |

**The data in Figure 13. Positioning trajectories and pose truths of different simultaneous localization and mapping algorithms under Sequence 6**

| Real coordinate | LOAM | LIO-Mapping | Ours | LeGO-LOAM | LINS | LIO-SAM |
| --- | --- | --- | --- | --- | --- | --- |
| (-150,0) | (-150,-8) | (-150,0) | (-150,0) | (-150,0) | (-150,0) | (-150,0) |
| (-50,-50) | (-50,-60) | (-50,-58) | (-50,-50) | (-50,0) | (-50,-26) | (-50,-38) |
| (50,-100) | (50,-105) | (50,-103) | (50,-100) | (50,-84) | (50,-96) | (50,-113) |
| (150,-300) | (150,-260) | (150,-304) | (150,-300) | (150,-294) | (150,-294) | (150,-304) |

**The data in Figure 14. Comparison results of error between indoor running path and theoretical path of the robot dog**

**(a) Comparison of actual and theoretical paths**

| Theoretical path | Error | | |
| --- | --- | --- | --- |
|  | Monocular visual fusion IMU | Multi-sensor fusion | Pure vision |
| (0.90,2.10) | 0.03 | 0.01 | 0.05 |
| (0.95,2.20) | 0.06 | 0.00 | 0.04 |
| (1.00,2.30) | 0.04 | 0.02 | 0.08 |
| (1.05,2.40) | 0.02 | 0.01 | 0.06 |
| (1.10,2.50) | 0.01 | 0.02 | 0.04 |
| (1.15,2.60) | 0.03 | 0.00 | 0.05 |
| (1.20,2.70) | 0.02 | 0.00 | 0.06 |

**(b) Actual and theoretical path errors**

| Theoretical path | Error | | |
| --- | --- | --- | --- |
|  | Monocular visual fusion IMU | Multi-sensor fusion | Pure vision |
| (0,3) | 0.06 | 0.03 | 0.13 |
| (25,1) | 0.08 | 0.01 | 0.15 |
| (50,2) | 0.04 | 0.02 | 0.12 |
| (75,3) | 0.10 | 0.01 | 0.09 |
| (100,0) | 0.02 | 0.03 | 0.08 |
| (125,15) | 0.06 | 0.02 | 0.13 |
| (150,12) | 0.05 | 0.00 | 0.14 |
| (175,6) | 0.04 | 0.04 | 0.11 |
| (200,6) | 0.08 | 0.06 | 0.10 |

**The data in Figure 15. The result of the deviation of the angle axis between the RD and the three-dimensional point cloud coordinates during the actual movement process**

| X (m) | Error (°) | | |
| --- | --- | --- | --- |
|  | Monocular visual fusion IMU (x,y,z) | Research methods (x,y,z) | Pure vision (x,y,z) |
| 0 | (-0.01,-0.13,-89.4) | (0.00,0.00,-90.0) | (-0.06,0.04,-89.1) |
| 10 | (-0.14,-0.25,-89.1) | (0.06,0.11,-90.0) | (-0.15,0.64,-89.2) |
| 20 | (-0.86,-0.36,-89.2) | (-0.46,0.62,-89.8) | (-0.74,0.89,-89.3) |
| 30 | (-0.62,-0.15,-90.0) | (-0.85,0.21,-89.5) | (-0.65,0.21,-89.2) |
| 40 | (-0.51,-0.08,-89.7) | (-0.24,0.19,-89.8) | (-0.34,0.33,-89.8) |
